# Members of chitin synthase family in *Metarhizium acridum* differentially affect fungal growth, stress tolerances, cell wall integrity and virulence

**DOI:** 10.1371/journal.ppat.1007964

**Published:** 2019-08-28

**Authors:** Junjie Zhang, Hui Jiang, Yanru Du, Nemat O. Keyhani, Yuxian Xia, Kai Jin

**Affiliations:** 1 School of Life Sciences, Chongqing University, Chongqing, People’s Republic of China; 2 Chongqing Engineering Research Center for Fungal Insecticide, Chongqing, People’s Republic of China; 3 Key Laboratory of Gene Function and Regulation Technologies under Chongqing Municipal Education Commission, Chongqing, PR China; 4 Department of Microbiology and Cell Science, University of Florida, Gainesville, Florida, United States of America; University of Melbourne, AUSTRALIA

## Abstract

Chitin is an important component of the fungal cell wall with a family of chitin synthases mediating its synthesis. Here, we report on the genetic characterization of the full suite of seven chitin synthases (*MaChsI-VII*) identified in the insect pathogenic fungus, *Metarhizium acridum*. Aberrant distribution of chitin was most evident in targeted gene knockouts of *MaChsV* and *MaChsVII*. Mutants of *MaChsI*, *MaChsIII*, *MaChsIV* showed delayed conidial germination, whereas Δ*MaChsII* and Δ*MaChsV* mutants germinated more rapidly when compared to the wild-type parent. All *MaChs* genes impacted conidial yield, but differentially affected stress tolerances. Inactivation of *MaChsIII*, *MaChsV*, *MaChsVII* resulted in cell wall fragility, and Δ*MaChsV* and Δ*MaChsVII* mutants showed high sensitivity to Congo red and calcofluor white, suggesting that the three genes are required for cell wall integrity. In addition, Δ*MaChsIII* and Δ*MaChsVII* mutants showed the highest sensitivities to heat and UV-B stress. Three of seven chitin synthase genes, *MaChsIII*, *MaChsV*, *MaChsVII*, were found to contribute to fungal virulence. Compared with the wild-type strain, Δ*MaChsIII* and Δ*MaChsV* mutants were reduced in virulence by topical inoculation, while the Δ*MaChsVII* mutant showed more severe virulence defects. Inactivation of *MaChsIII*, *MaChsV*, or *MaChsVII* impaired appressorium formation, affected growth of *in insecta* produced hyphal bodies, and altered the surface properties of conidia and hyphal bodies, resulting in defects in the ability of the mutant strains to evade insect immune responses. These data provide important links between the physiology of the cell wall and the ability of the fungus to parasitize insects and reveal differential functional consequences of the chitin synthase family in *M*. *acridum* growth, stress tolerances, cell wall integrity and virulence.

## Author summary

The fungal cell wall is a dynamic and flexible organelle that modulates the interaction of the pathogen with its host and acts as a critical recognition and evasion interface with host defenses. Chitin is a hallmark constituent of the fungal cell wall and all fungi contain multiple chitin synthase (*Chs*) genes. However, systematic characterization of chitin synthase genes has not yet been reported in entomopathogenic fungi. By using the insect pathogen *Metarhizium acridum* as a model, we performed a systematic genetic analysis of the seven member *Chs* family (*ChsI-VII*) in the insect pathogenic fungus. Construction of strains bearing targeted single gene mutations revealed differential contributions of specific *Chs* genes to growth, cell wall integrity, and stress responses. In addition, we revealed that three chitin synthase genes *MaChsIII*, *MaChsV* and *MaChsVII* were shown to be important for fungal appressorium formation and evasion of insect cellular and/or humoral defenses, promoting the fungal dimorphic transition to the production of hyphal bodies that occurs within hosts, and ultimately to virulence. These data provide new insights into the roles of *Chs* genes and chitin as critical components affecting fungal membrane structure and function.

## Introduction

The fungal cell wall is a dynamic and flexible organelle that modulates the interaction of the organism with its environment and, in the case of pathogens, acts as a critical recognition and evasion interface with host defenses [1]. Chitin, a homopolymer of β-(1*/*4)–linked *N*-acetylglucosamine, is a basic component of the fungal cell wall, and its synthesis required for normal hyphal growth and spore production [2]. Chitin fibrils and cross-linked proteins help shape and maintain the overall mechanical strength of the fungal cell, contributing to the environmental survival and virulence in pathogenic fungi [3–6]. Fungal chitin biosynthesis is catalyzed by a family of chitin synthases grouped into seven classes (I to VII) based on amino acid homology, with variation seen in the number of chitin synthase (*Chs*) genes seen in different fungi [2, 7]. In the budding yeast *Saccharomyces cerevisiae*, three *Chs* genes *Chs1* (class I), *Chs2* (class II) and *Chs3* (class IV) are present [7], whereas *Chs* III, V, VI and VII are found only in filamentous fungi [8]. Filamentous fungi often contain 6–10 *Chs* genes, e.g. the plant pathogen *Ustilago maydis* contains eight *Chs* genes (*Chs1-Chs7* and *Mcs1*) in its genome [9].

In *S*. *cerevisiae*, the three *Chs* genes have distinct functional roles in cell wall expansion, septum formation, and budding [10, 11]. *Chs1* (class I) is responsible for cell wall repair and regeneration during division as mother and daughter cells separate. *Chs2* (class II) synthesizes chitin that is localized to the primary septum during its formation [12]. *Chs3* (class IV) catalyzes the synthesis of the chitin ring found at the base of an emerging bud. *Chs3* (class IV) appears to be the most catalytically active enzyme in yeast, involved in most chitin synthesis, whereas *Chs1* (class I) and *Chs2* (class II) synthesize relatively small amounts of chitin [13]. In the filamentous fungus, *Aspergillus nidulans*, deletion of *ChsA* (class II), *ChsC* (class III), or *ChsD* (class IV) individually did not lead to any obvious phenotypes, whereas deletion of *ChsB* (class I) resulted in defects in hyphal growth [14–16].

Variations in the functions of *Chs* homologues belonging to the same class in different fungi had also been reported. In the plant pathogen, *Ustilago maydis*, inactivation of *Chs1* (class III) had no obvious effects on either growth or virulence [9]. However, in the rice blast fungus, *Magnaporthe oryzae*, inactivation of the homologous *Chs1* (class III) resulted in significantly reduced virulence towards plant hosts [6, 17]. In the human pathogen, *A*. *fumigatus*, the homologous *ChsG* (class III) mutant showed no defects in virulence, hypha morphology, or sensitivity to cell wall disturbing agents [18]. These data indicate that important functional divergences between homologous family members in different fungi appear to have occurred. Thus, in *A*. *fumigatus*, of eight *Chs* genes (*ChsA-F*, *csmA* and *csmB*) that have been identified, two MMD (myosin motor domain)-containing chitin synthase genes, *csmA* (class V) and *csmB* (class VII) have been shown to play important roles in fungal virulence [18–20]. In *U maydis*, *Chs7* (class IV), *mcs1* (class V) and *Chs6* (class VII) have been shown to impact fungal virulence, whereas the nature of the infection process affected by different *Chs* mutants has been shown to differ [9, 21, 22]. In *M*. *oryzae*, three of the seven chitin synthase genes identified have been implicated in contributing to fungal virulence, two of which belong to different classes than the *Chs* genes involved in *U*. *maydis* infection [6, 23, 24].

Little, however, is known about the function of *Chs* genes in other filamentous fungi, especially beyond plant and animal pathogens. Insect pathogenic fungi are emerging as a novel model system that can be used to examine unique aspects of fungal development and virulence. Genome sequences of species of the *Beauveria* and *Metarhizium* genera are available [25, 26], along with molecular methods for genetic manipulation, and, especially compared to animal pathogens, insect hosts are inexpensive and can readily be obtained in large quantities for experimental use. Infection of insect hosts by entomogenous fungi involves conidial (spore) attachment and germination on the host (epi) cuticle, followed by formation of exoskeleton penetrating structures (e.g. appressoria) [27, 28]. The penetrating hyphae reach the insect hemocoel where they undergo a dimorphic transition to the production of yeast-like hyphal bodies that disseminate throughout the hemolymph, having evolved the ability to overcome/evade insect immune defenses [29, 30]. The fungus then grows outwards from within the insect body, ultimately killing the host and sporulating on the cadaver [31]. Successful infection depends on the ability of the fungus to overcome host antimicrobial (antifungal) responses. The insect innate immune system can recognize fungal-specific components of the cell wall [32–34] and/or sense various fungal virulence factors [34–36]. In response to fungal infections, several host immune defenses can be activated including, (i) melanization, mediated by the prophenoloxidase pathway [37], (ii) cellular immune defenses, such as the phagocytosis mediated by hemocytes [38] and nodulation [39], and (iii) humoral immune defenses, that include production of antimicrobial peptides (AMPs) via the action of the Toll and/or immune deficiency (Imd) pathways [40, 41]. Whereas most *Beauveria* and *Metarhizium* species are broad host range cosmopolitan pathogens, *M*. *acridum* displays a narrower host range towards acridids (grasshoppers and locusts) [26]. Here, we report on the systematic genetic dissection of the seven-member chitin synthase family (*MaChsI*-*VII*) in *M*. *acridum* via characterization of target single knockout mutants of each gene. Our findings demonstrate that individual *MaChs* genes played differential roles in fungal growth, stress responses, cell wall integrity and virulence. These data provide important links between chitin involved in fungal membrane and cell wall structure and hyphal growth, appressorium development, and fungal dimorphic transition, impacting the ability of the fungus to respond to abiotic stress as well as to successfully infect and parasitize insect hosts.

## Results

### Identification, expression analyses, and construction of targeted gene knockout mutants of the 7-member chitin synthase gene family in *M*. *acridum*

**Fig 1:**
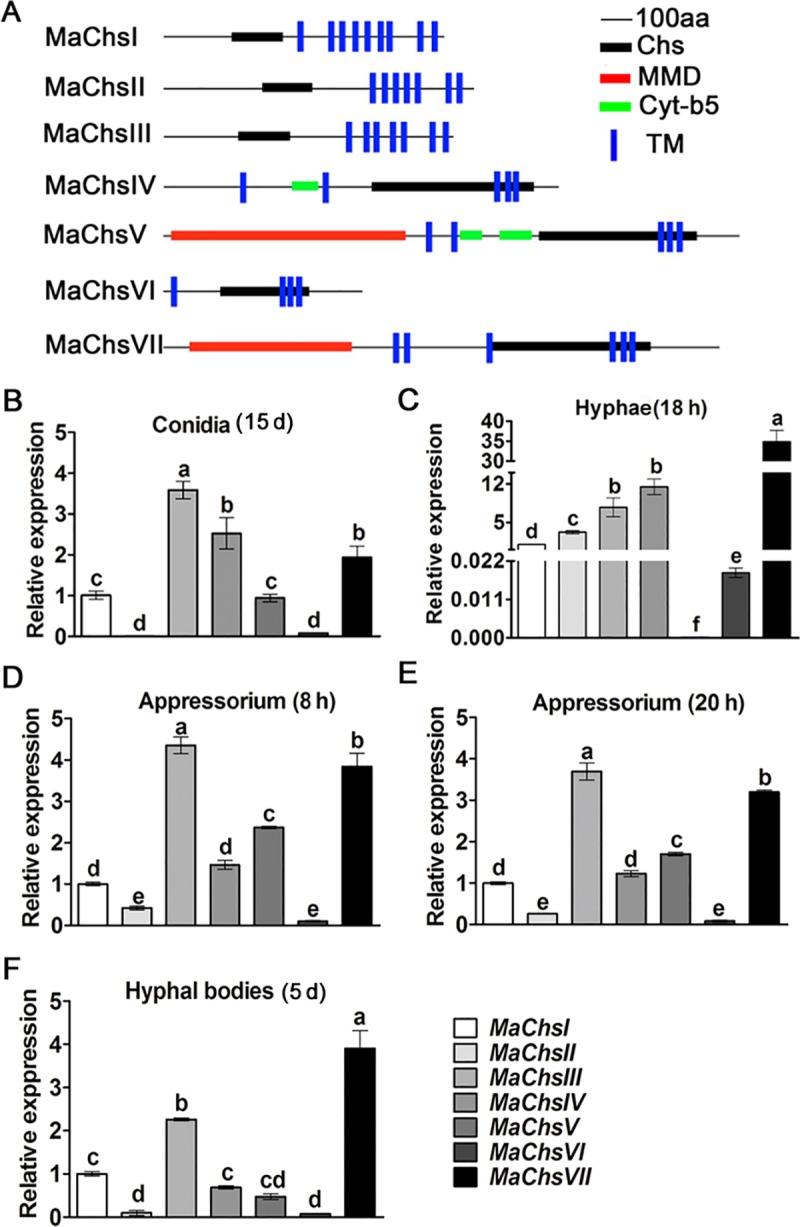
Domain organization and expression profile of the seven chitin synthases in *M*. *acridum*. (A) Domain structures of the seven chitin synthases in *M*. *acridum*. Chs, chitin synthase domain; MMD, myosin motor domain; Cyt-b5, cytochrome b5-like heme/steroid binding domain; TM, transmembrane domain. (B) The expression levels of seven *Chs* genes in conidia. Conidia harvested from 15-day-old 1/4 SDAY cultures. (C) The expression levels of seven *Chs* genes in hyphae. Hyphae harvested from 18-hour-old 1/4 SDAY cultures. (D) The expression levels of seven *Chs* genes during germination on the locust hind wings. Samples were collected at 8 h after inoculation on the locust hind wings. (E) The expression levels of seven *Chs* genes during appressorium formation on the locust hind wings. Samples were collected at 20 h after inoculating on the locust hind wings. (F) The expression levels of seven *Chs* genes in hyphal bodies. Hyphal bodies were harvested from locust hemolymph at 5 d after inoculation. Expression profiles of seven *MaChs* genes assayed by qRT-PCR. The expression level of *MaChsI* was arbitrarily set to 1. Standard errors were determined with data from three independent replicates. Different lowercase letters on bars denote significant difference, *P* < 0.05.

Sequence analysis showed that the *M*. *acridum* genome includes seven predicted chitin synthase genes [26]. A phylogenetic tree using functionally characterized Chs proteins from other fungi was constructed using the neighbor-joining method as detailed in the Materials and methods section. The chitin synthase genes in *M*. *acridum* were named as *MaChsI*-*VII* in accordance with their placement among orthologs (S1 Fig). All the *M*. *acridum* chitin synthase genes had multiple transmembrane (TM) domains apart from the active site chitin synthase domain. *MaChsIV* and *MaChsV* had one cytochrome b5-like heme/steroid-binding domain (Cyt-b5) upstream from Chs domain, and *MaChsV* and *MaChsVII* contained myosin motor domains (MMD) at their N-termini (Fig 1A).

The expression levels of the *MaChs* genes were examined by real time reverse transcription PCR (qRT-PCR) in conidia, hyphae, infection structures (appressoria), and in cells produced inside the host during infection (hyphal body). In conidia, *MaChsIII* and *MaChsIV* were the most highly expressed, followed by *MaChsVII* > *MaChsI* and *MaChsV*, with little to no expression of *MaChsII* or *MaChsVI* seen (Fig 1B). In hyphae, *MaChsVII* showed the highest relative expression > *MaChsIV* > *MaChsIII* > *MaChsII* > *MaChsI*, with little expression of *MaChsVI* and no expression of *MaChsV* seen (Fig 1C). During appressorium formation (at both 8 and 20 h, p.i.), *MaChsIII* and *MaChsVII* were the most highly expressed followed by *MaChsV* then *MaChsIV* > *MaChsI* > *MaChsII* > *MaChsVI* (Fig 1D and 1E). In contrast, *MaChsVII* showed the highest relative expression in hyphal bodies > *MaChsIII* > *MaChsI* > *MaChsIV* and *MaChsV*, with little expression of either *MaChsII* or *MaChsVI* seen in these cells (Fig 1F).

In order to probe the biological functions of the *M*. *acridum Chs* gene family, targeted gene disruption mutants as well as complemented strains were constructed via homologous recombination as detailed in the Materials and methods section. Gene disruption vectors were constructed to replace genomic target regions with a 900 bp cassette of phosphinothricin resistance marker (*bar*). Complemented strains were made via ectopic insertion with promoter regions. Initial screening of putative transformants was performed by PCR followed by verification using Southern blotting (S2–S8 Figs). Unless otherwise noted, complemented strains of each respective *MaChs* gene were identical to wild type in phenotypes examined.

### Disruption of *MaChs* genes affects fungal vegetative growth and hypha morphology

**Fig 2:**
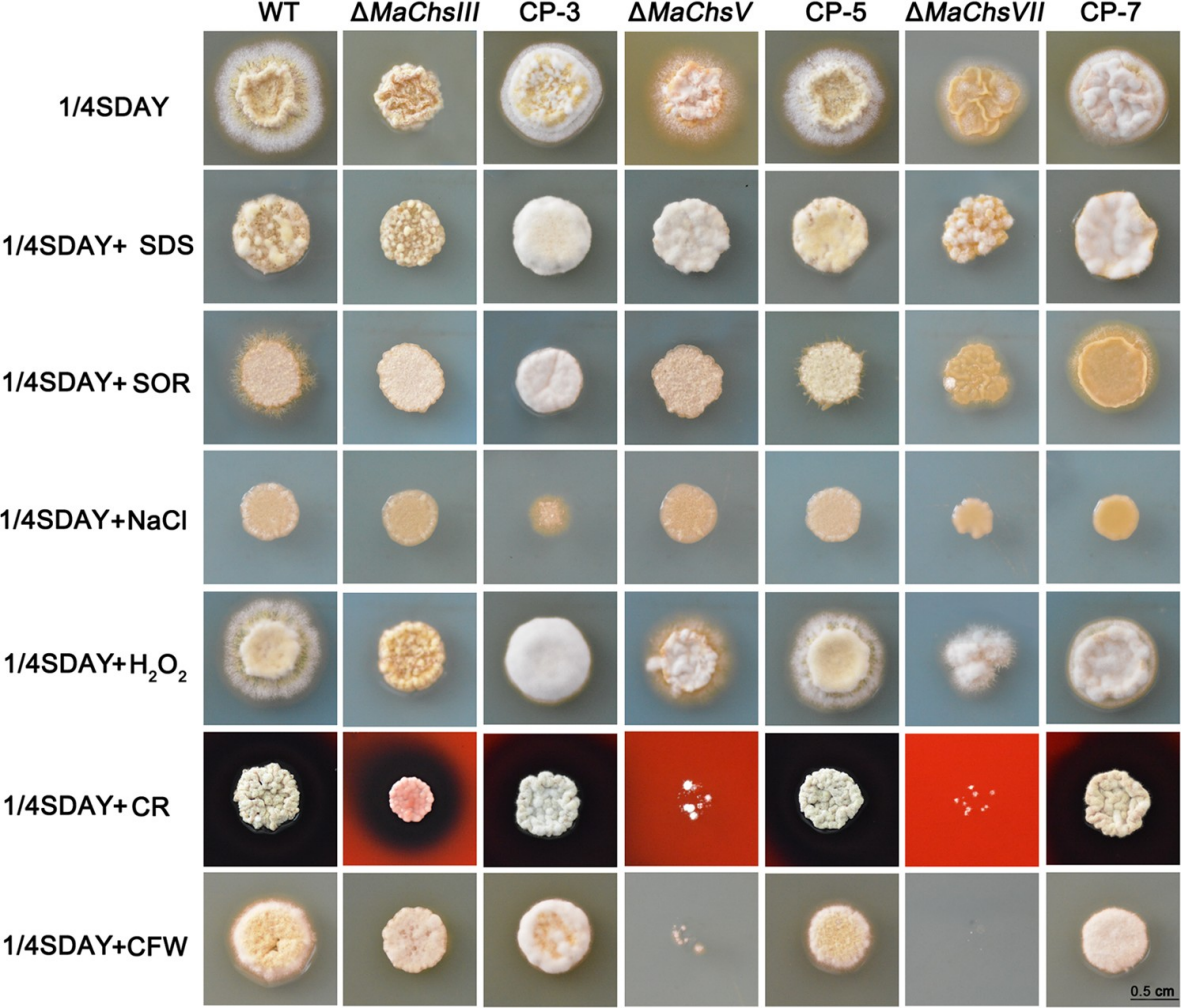
Colony morphology of the wild-type strain and the Δ*MaChsIII*, Δ*MaChsV*, Δ*MaChsVII* mutants. Colony morphology of the *MaChs* deletion mutants on 1/4 SDAY or 1/4 SDAY supplemented with 0.1% SDS (sodium dodecyl sulfate), 1.5 mol l^-1^ Sorbitol, 0.5 mol l^-1^ NaCl, 500 μg ml^-1^ CR (Congo red), 50 μg ml^-1^ CFW (calcofluor white), 6 mmol l^-1^ H_2_O_2_ at 28°C. The fungal colonies were photographed after 5 d of incubation. Bar scale = 0.5 cm.

**Fig 3:**
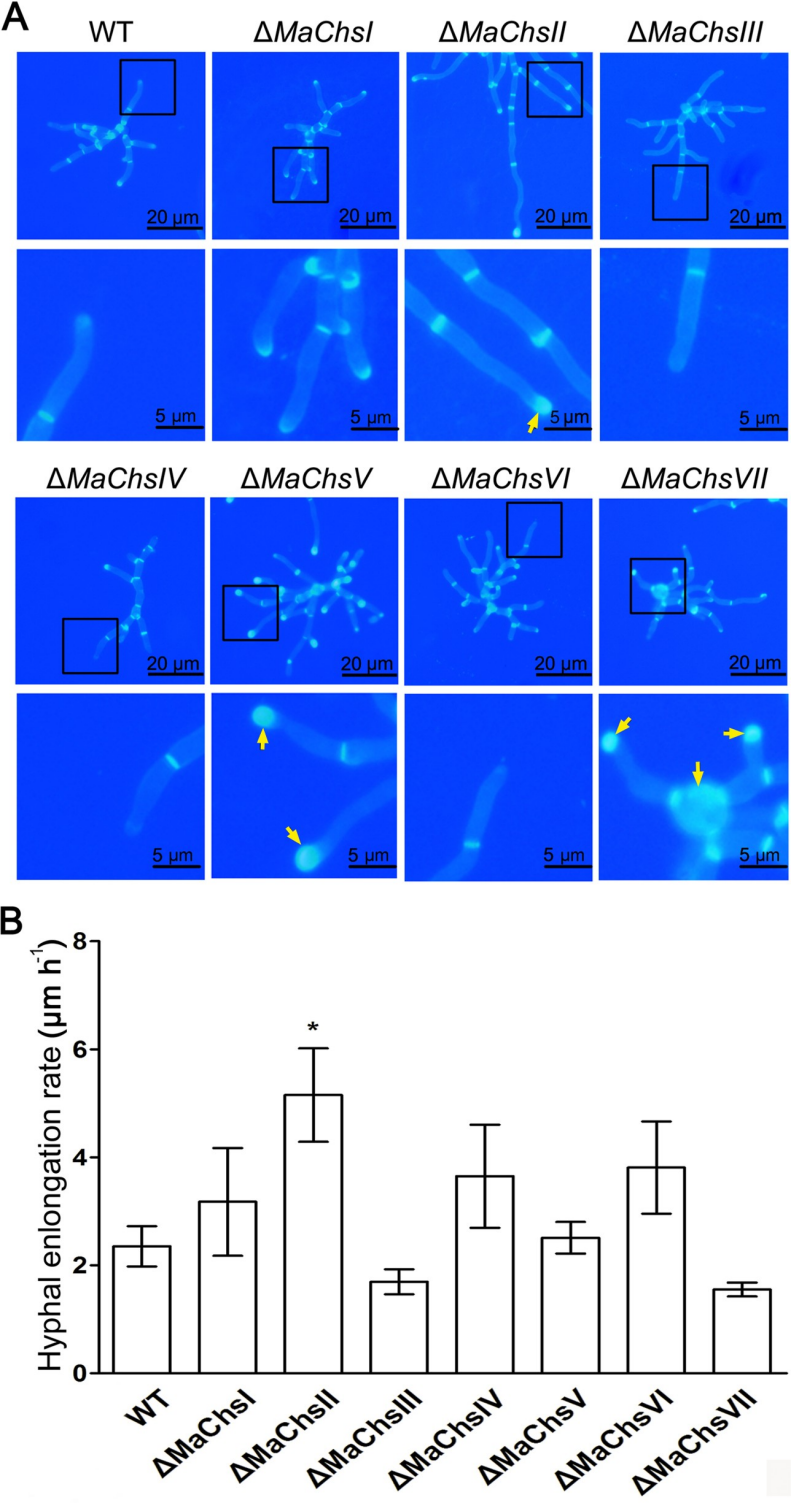
Growth and hyphal morphology of *Chs* mutants. (A) Hyphal morphology of wild type and Δ*MaChs* mutants on 1/4 SDAY. Cell wall chitin of wild type and Δ*MaChs* mutant hyphae were observed staining with calcofluor white on a cover glass by fluorescence microscopy. Arrows indicate the site of chitin accumulation. (B) Hyphal growth rates of wild type and Δ*MaChs* mutants on 1/4 SDAY. A single asterisk above bars denotes significant difference, *P* < 0.05. Error bars indicate standard errors of three trials.

On 1/4 strength Sabouraud dextrose agar (1/4 SDAY) medium, no growth difference was observed for the Δ*MaChsI*, Δ*MaChsII*, Δ*MaChsIV* and Δ*MaChsVI* mutants as compared to the wild-type strain (S9 Fig). In contrast, the Δ*MaChsIII*, Δ*MaChsV* and Δ*MaChsVII* mutants displayed aberrant colony phenotypes and grew markedly slower than the wild type and the corresponding complemented strains (Fig 2). The Δ*MaChsIII* mutant produced a compact colony with limited radial growth. This mutant showed no appreciable additional phenotypes on media supplemented with SDS, sorbitol, high salt (NaCl), H_2_O_2_, or calcofluor white, with only moderate impairment of growth seen in the presence of Congo red. Similarly, minor phenotypes were seen for the Δ*MaChsV* and Δ*MaChsVII* strains undermost conditions, with the exception of severe sensitivity to Congo red and calcofluor white. No significant phenotypes were seen for Δ*MaChsI*, *II*, *IV*, *VI* mutants under similar conditions. Only a small effect was seen in the overall vegetative growth of the Δ*MaChsVII* strain grown in liquid 1/4 SDY for 3 d, with no significant differences seen for any of the other mutants (S10 Fig). In order to probe any effects on hyphal morphology, hyphae were examined microscopically after staining for chitin using calcofluor white. A number of distinct aberrant phenotypes in different *Chs* mutants were noted (Fig 3A). Hyphae from the Δ*MaChsII* mutant were more elongated than wild-type cells, especially at the apical cell regions, with greater chitin deposition seen at hyphal tips, the latter a phenomenon seen to a lesser extent in the *MaChsI* mutant (arrows in Fig 3A). Although overall hyphal morphology for the Δ*MaChsV* mutant appeared unaffected, enhanced chitin accumulation was seen at septae with dramatically increased chitin accumulation seen at the hyphal tips (arrows in Fig 3A). The *MaChsVII* mutant showed swelling or ballooning of intercalary cells, with distortions and chitin accumulation (mainly at tips, arrows in Fig 3A). Little to no noticeable changes in calcofluor white staining were seen for the *MaChsIII*, *IV*, or *VI* mutants. In addition, measurement of hyphal elongation rates on 1/4 SDAY confirmed that the elongation (growth) for the Δ*MaChsII* mutant was significantly faster (*P* < 0.05) than wild type, while no significant differences were found between the wild type and other *MaChs* mutants (Fig 3B).

### Specific *MaChs* genes affected conidial germination

**Fig 4:**
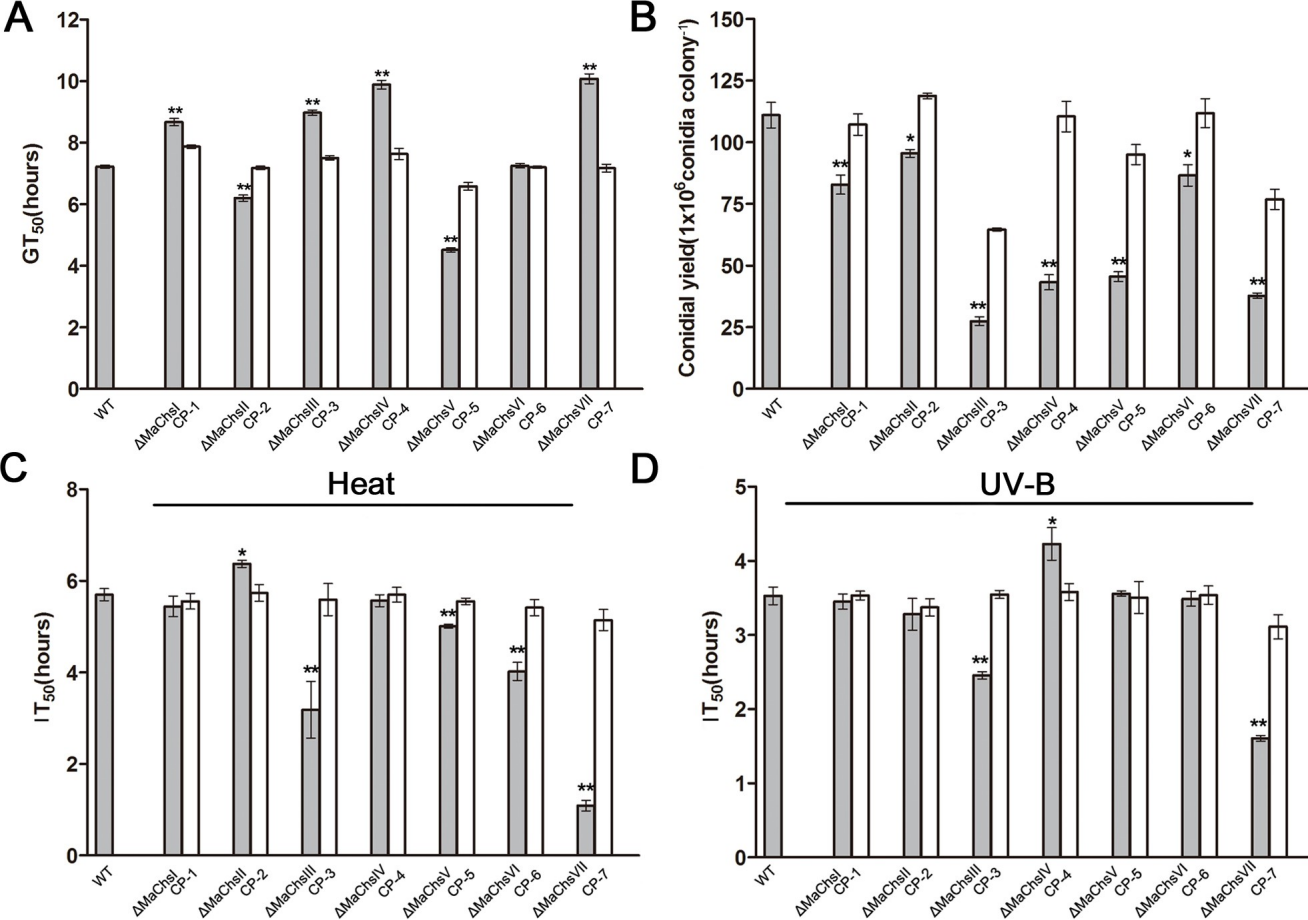
Measurement of conidial germination and yield, and fungal stress responses. (A) Germination time values for 50% germination (GT_50_) of conidia of wild type and Δ*MaChs* mutants. (B) Conidial yields of wild type and Δ*MaChs* mutants on 1/4 SDAY at 28°C for 15 d. (C) Inhibition time values for 50% germination (IT_50_) of wild type and Δ*MaChs* mutants by heat shock. IT_50_ estimates from the fitted trends of the conidial germination after exposure for 2, 4, and 6 h to of 45°C heat shock. (D) Inhibition time values for 50% germination (IT_50_) of wild type and Δ*MaChs* mutants by UV-B irradiation. IT_50_ estimates from the fitted trends of the conidial germination after exposure for 2, 4, and 6 h to UV-B irradiation of 1350 mW/m^2^. A single asterisk above bars denotes significant difference, *P* < 0.05; double asterisks above bars denote significant difference, *P* < 0.01. Error bars indicate standard errors of three trials.

The time required for 50% of the conidia to germinate (GT_50_) of the Δ*MaChsI*, Δ*MaChsIII*, Δ*MaChsIV* and Δ*MaChsVII* mutants were significantly longer (*P* < 0.05, i.e. slower germination) than the wild type. In contrast, the GT_50_ of the Δ*MaChsII* and Δ*MaChsV* mutants were significantly shorter (*P* < 0.05), i.e. they germinated faster than the wild type (Fig 4A and S11 Fig). Quantification of conidial yield revealed that all *MaChs* mutants produced significantly (*P* < 0.05) less conidia than the wild type, with the most notable reductions in spore production seen in the Δ*MaChsIII*, Δ*MaChsIV*, Δ*MaChsV* and Δ*MaChsVII* mutants, with approximately 60% less conidia produced by these latter strains than the wild-type strain after 15 d of incubation. These results indicated that chitin synthesis plays an important role in fungal conidiation (Fig 4B).

The germination rates of the mutant strains after exposure to UV-B radiation or incubation at 45°C were also examined as a function of exposure time. The results showed that the germination rates of all strains decreased with increasing of treatment time. For heat shock (45°C), the exposure time required for 50% inhibition of germination (IT_50_) of the wild type was significantly higher (*P* < 0.05) than that of the Δ*MaChsIII*, Δ*MaChsV*, Δ*MaChsVI* and Δ*MaChsVII* mutants, while lower than that for the Δ*MaChsII* mutant (Fig 4C). These results indicated that inactivation of *MaChsIII*, *MaChsV*, *MaChsVI* and *MaChsVII* increased fungal sensitivity to heat, while disruption of *MaChsII* decreased fungal sensitivity to heat. With respect to exposure to UV-B irradiation, the IT_50_ of the wild type was significantly higher than that of the Δ*MaChsIII* and Δ*MaChsVII* mutants, while lower than that of the Δ*MaChsIV* mutant, with no significant differences seen for the other *MaChs* mutants (Fig 4D). These results indicated that inactivation of *MaChsIII* and *MaChsVII* increased fungal sensitivity to UV-B treatment, while disruption of *MaChsIV* decreased fungal sensitivity to UV-B treatment.

### Effect of disruption of *MaChs* genes on cell wall structure and composition

**Fig 5:**
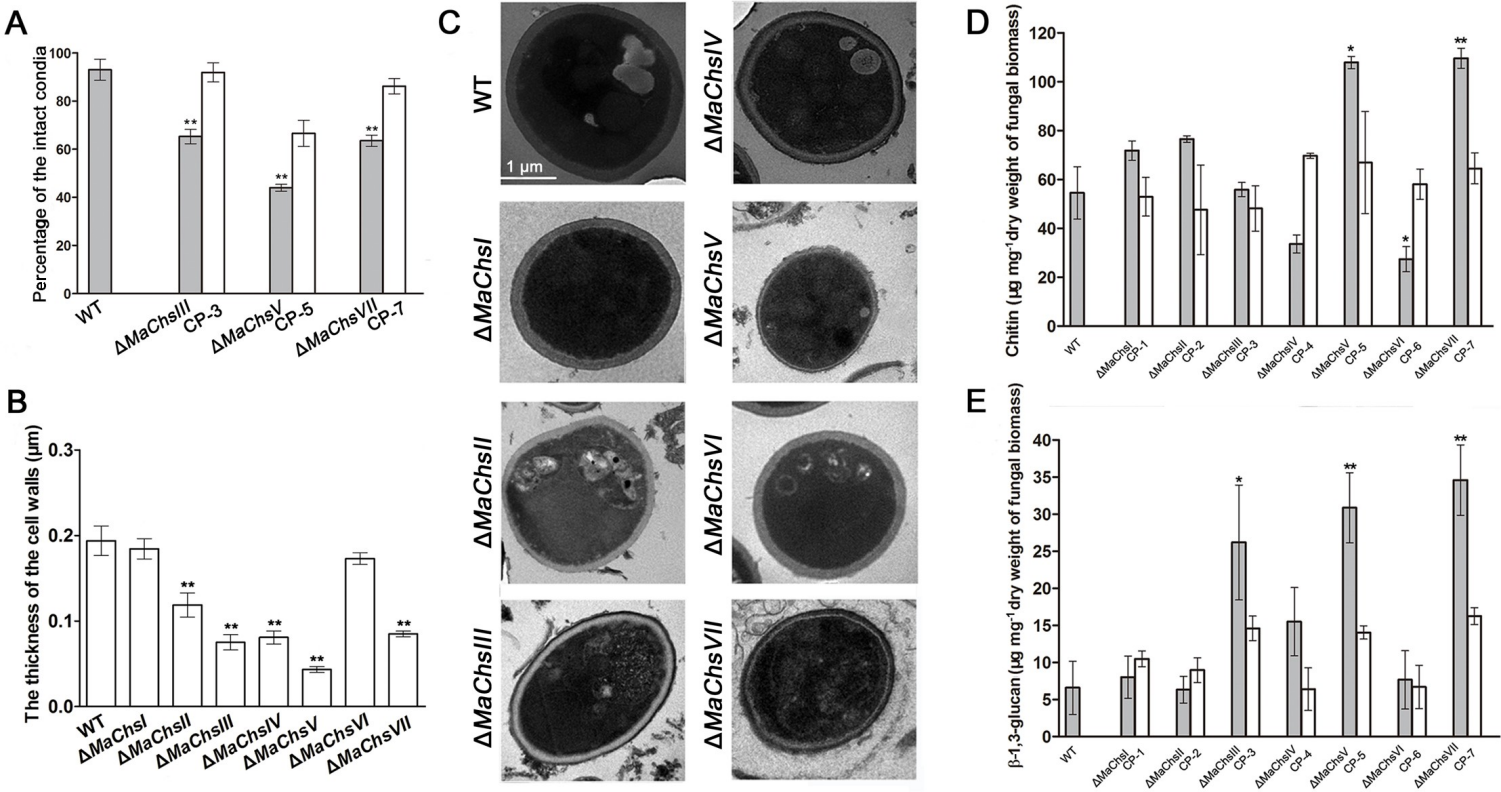
Cell wall structure and composition of wild type and Δ*MaChs* mutants. (A) Measurements of cell wall integrity for the wild type and Δ*MaChsIII*, Δ*MaChsV*, Δ*MaChsVII* mutants. (B) The cell wall thickness of the wild type and *MaChs* mutants. (C) Transmission electron micrographs (TEM) images for the cell wall distortions seen in conidia produced by the wild type and *MaChs* mutants. Bar scale = 1 μm. (D) Chitin content as determined by acid hydrolysis of fungal cell wall. (E) β-1,3-glucan determined via quantification of the alkali-insoluble fraction of the cell wall. Samples were vegetative hyphae harvested from 3-day-old liquid 1/4 SDY cultures. A single asterisk above bars denotes significant difference, *P* < 0.05; double asterisks above bars denote significant difference, *P* < 0.01. Error bars indicate standard errors of three trials.

To investigate the contributions of individual *M*. *acridum* chitin synthases on cell wall structure, the ability of conidia derived from the various mutant strains to resist distortion during application of a centrifugal force was investigated as detailed in the Materials and methods section, the results revealed that ~30% to 50% of the conidia derived from the Δ*MaChsIII*, Δ*MaChsV* and Δ*MaChsVII* mutants were distorted or broken, whereas ~90% of the conidia of the wild type and remaining mutant (Δ*MaChsI*, *II*, *IV*, *VI*) strains were still intact (Fig 5A). In addition, examination of the cell walls of conidia by TEM revealed significant changes in cell wall thickness, with 1.63- (*P* < 0.01), 2.58- (*P* < 0.01), 2.40- (*P* < 0.01), 4.48- (*P* < 0.01) and 2.28- (*P* < 0.01) fold decreases in cell wall thickness seen for the Δ*MaChsII*, Δ*MaChsIII*, Δ*MaChsIV*, Δ*MaChsV* and Δ*MaChsVII* mutants, respectively, as compared to the wild type and each respective complemented strain (Fig 5B and 5C).

In order to examine the extent to which altered chitin synthesis in any of the mutants affect fungal cell wall carbohydrate components, the overall chitin, β-1,3-glucan, and mannoprotein contents in the cell walls of all of the strains were determined. Loss of either the *MaChsI*, *MaChsII*, or *MaChsIV* chitin synthases did not significantly alter chitin, β-1,3-glucan, or mannoprotein levels in the cells as compared to the wild type parent. In contrast, significant changes in these cell wall components were seen in mutants of the *MaChsIII*, *V*, *VI*, and *VII* genes. Chitin levels were reduced by 37% in the Δ*MaChsVI* mutant compared to the wild type (Fig 5D), whereas both chitin and β-1,3-glucan levels in the Δ*MaChsV* and Δ*MaChsVII* mutants cell walls were significantly higher than those of wild-type cells (Fig 5E). Total mannoprotein content was reduced slightly (~10%) in the Δ*MaChsVI* mutant but was increased (~20%) in the Δ*MaChsIII* mutant as compared to wild type levels (S12 Fig).

### The *MaChsIII*, *MaChsV* and *MaChsVII* genes are important for fungal virulence

**Fig 6:**
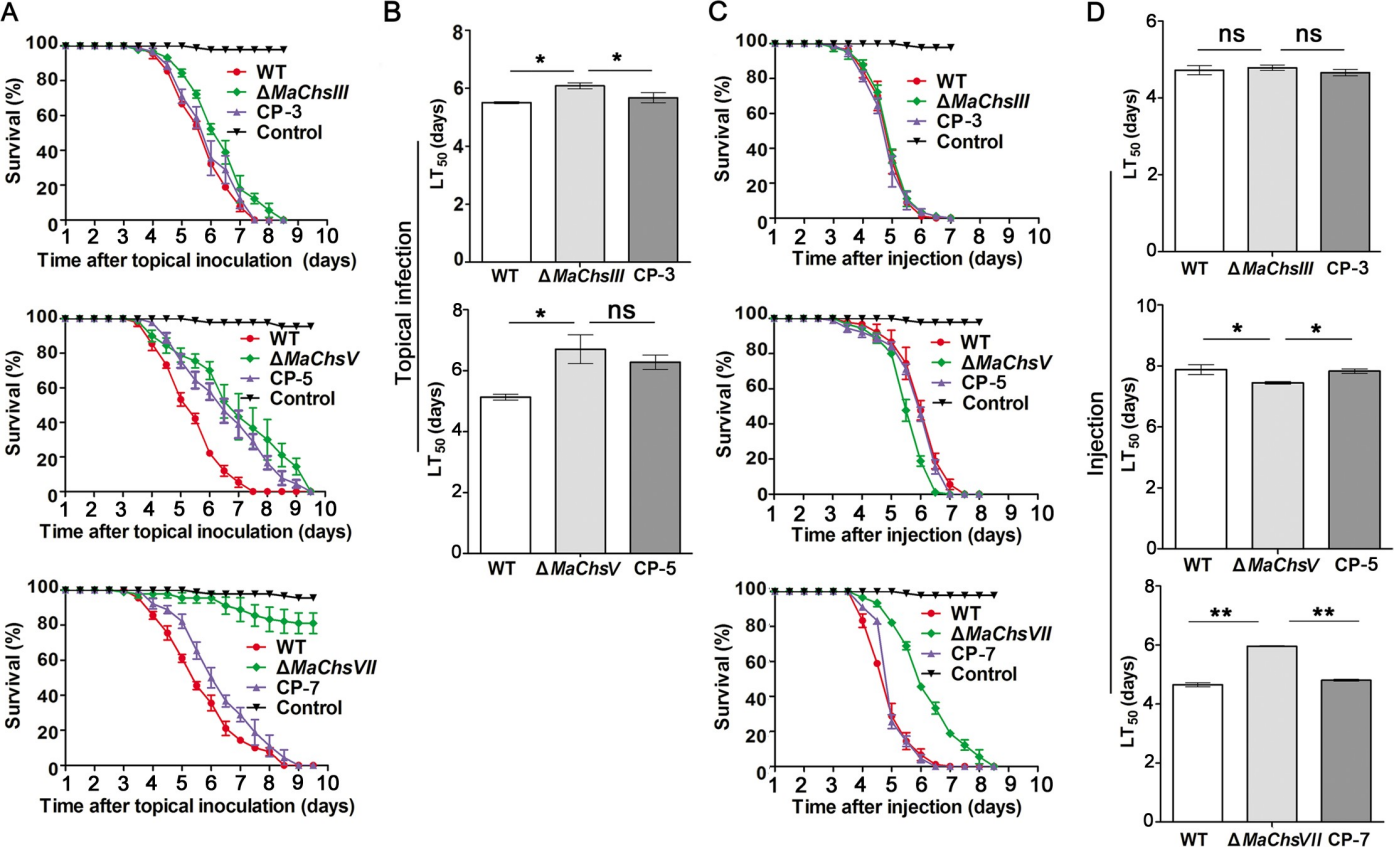
Effect of *MaChsIII*, *MaChsV* and *MaChsVII* mutants on the fungal virulence. (A) Survival of locusts after topical inoculation with 5 μl of 0.05% Tween-80 containing 1×10^8^ conidia ml^-1^ of wild type, Δ*MaChsIII*, Δ*MaChsV* and Δ*MaChsVII* mutants. (B) The mean 50% lethality time (LT_50_) by topical inoculation of wild type and Δ*MaChsIII*, Δ*MaChsV* mutants. (C) Survival of locusts after injection with 5 μl of sterile water containing 1×10^6^ conidia m^-1^ of wild type and Δ*MaChsIII*, Δ*MaChsV*, Δ*MaChsVII* mutants. (D) The mean 50% lethality time (LT_50_) by injection of wild type and Δ*MaChsIII*, Δ*MaChsV*, Δ*MaChsVII* mutants. A single asterisk above bars denotes significant difference, *P* < 0.05; double asterisks above bars denote significant difference, *P* < 0.01. ns indicates no significant difference, *P* > 0.05. Error bars indicate standard errors of three trials.

To determine contributions of the *MaChs* genes in fungal virulence, two types of bioassays were employed, namely, (1) topical inoculation requiring cuticle penetration and representing the natural route of parasitic infection, and (2) intrahaemocoel injection of conidia, thus bypassing the cuticle penetration stage, but requiring the fungus to retain the ability to evade host hemolymph defenses. In both topical inoculation and intrahaemocoel injection bioassays, no significant changes in virulence as measured by the mean lethal time to kill (LT_50_) were seen for the Δ*MaChsI*, Δ*MaChsII*, Δ*MaChsIV*, or Δ*MaChsVI* mutants (S13 Fig). However, the Δ*MaChsIII* and Δ*MaChsV* mutants exhibited significantly (*P* < 0.05) reduced virulence in topical inoculation assays (Fig 6A and 6B). Interestingly, disruption of *MaChsIII* had no effect when the conidia were directly injected into the host hemocoel, however for the *MaChsV* mutant a small, but significant (*P* < 0.05) increase in fungal virulence was seen in these assays (Fig 6C and 6D). Unlike the other *MaChs* mutants, the Δ*MaChsVII* mutant was essentially non-pathogenic when applied topically (Fig 6A) and significantly reduced (~81%, *P* < 0.01) in virulence by intrahaemocoel injection (Fig 6C and 6D). These data demonstrate that *MaChsIII* and *MaChsV* are both involved in fungal virulence during the normal infection via cuticle penetration, and *MaChsVII* play important roles in mediating virulence in *M*. *acridum*.

### *MaChsIII*, *MaChsV* and *MaChsVII* are involved in the conidial surface hydrophobicity, appressorium formation and fungal growth in insect hemolymph

**Fig 7:**
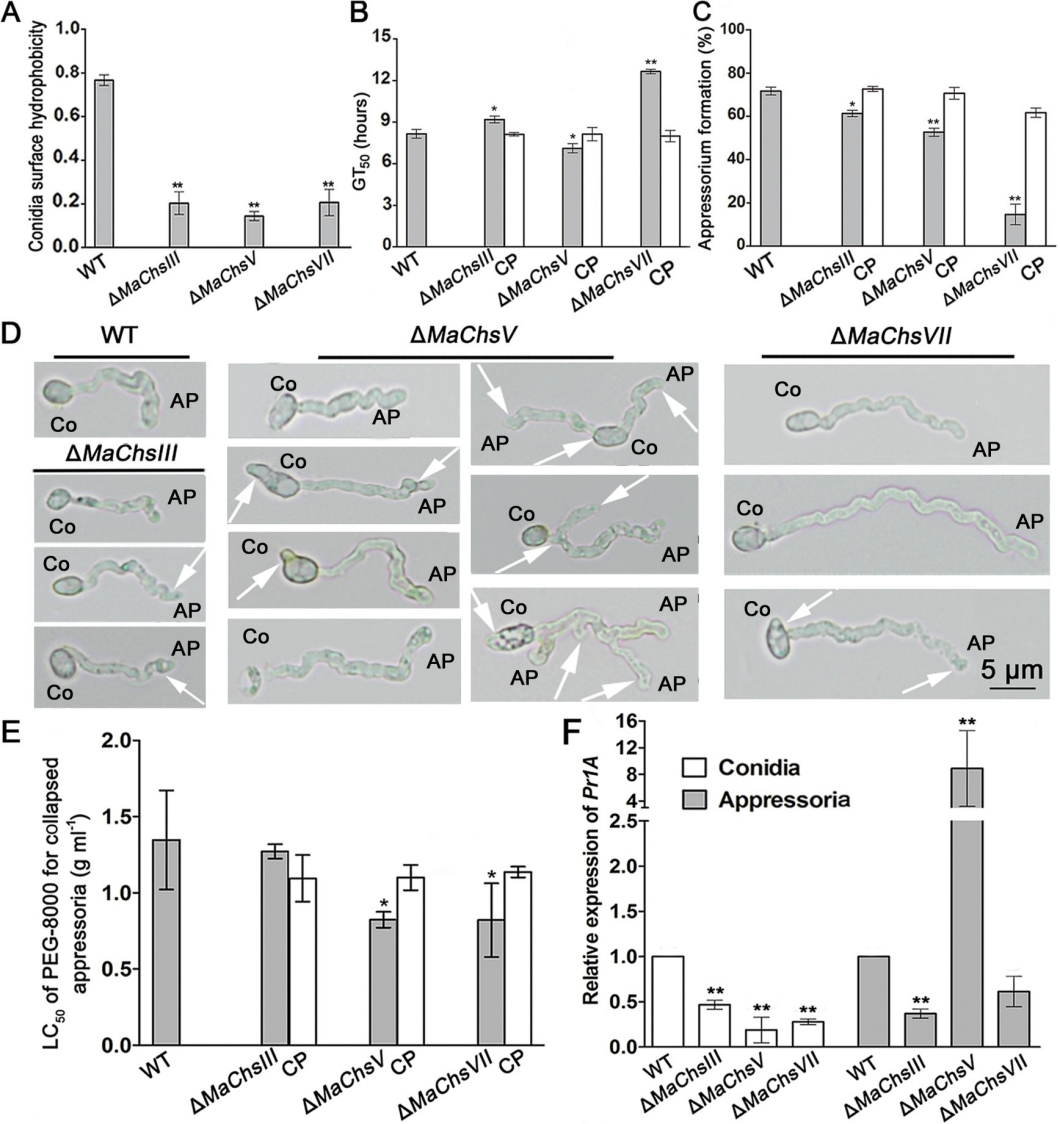
Conidial germination and appressorium formation on locust hind wings. (A) The hydrophobicity of conidia from wild type, Δ*MaChsIII*, Δ*MaChsV*, Δ*MaChsVII* mutants. (B) Germination time values for 50% germination (GT_50_) of wild type, Δ*MaChsIII*, Δ*MaChsV*, Δ*MaChsVII* mutants on locust hind wings. (C) Rates of appressorium formation in germinated conidia. (D) Appressorium morphology of wild type, Δ*MaChsIII*, Δ*MaChsV*, Δ*MaChsVII* mutants. White arrows represent the various defects of conidia and appressoria. (E) LC_50_ of PEG-8000 of collapsed appressoria. (F) The relative expression levels of *Pr1A* gene (GenBank accession number: AAV97788) in mature conidia and appressoria (after induced 30 h on locust wings) of wild type, Δ*MaChsIII*, Δ*MaChsV*, Δ*MaChsVII* mutants. A single asterisk above bars denotes significant difference, *P* < 0.05; double asterisks above bars denote significant difference, *P* < 0.01. Error bars indicate standard errors of three trials.

To further probe potential mechanisms underlying the reduced virulence seen in the Δ*MaChsIII* and Δ*MaChsV* mutants, and the near loss of pathogenicity in Δ*MaChsVII* mutant seen in topical inoculations of these mutants, conidial surface hydrophobicity, conidial germination and appressorium formation on locust hind wings were examined. Conidial hydrophobicity assays revealed significant (*P* < 0.01) reductions in spore hydrophobicity for the Δ*MaChsIII*, Δ*MaChsV* and Δ*MaChsVII* mutants as compared to the wild type, with little to no changes seen for the other mutants (Fig 7A and S14 Fig). The GT_50_ on locust wings of Δ*MaChsIII* and Δ*MaChsVII* mutants was significantly higher than the wild type, while the GT_50_ of the Δ*MaChsV* mutant was lower than the wild type (Fig 7B). Measurement of appressorium formation on locust wings revealed defects in the Δ*MaChsIII*, Δ*MaChsV* and Δ*MaChsVII* mutants as compared to the wild type and corresponding complemented strains. The percentage of appressoria forming from germinated conidia after 48 h of incubation was ~53, 61, and 15% for the Δ*MaChsIII*, Δ*MaChsV* and Δ*MaChsVII* mutants, respectively, whereas greater than 70% of the germinated wild-type conidia formed appressoria under identical conditions (Fig 7C). In addition to decreased appressorium formation, various morphological defects could be seen in *MaChs* mutants. Distortions, including smaller and deformed structures were seen for Δ*MaChsIII* appressoria, and irregular formations, i.e. a single conidium forming multiple appressoria, cell-expansion and abnormal conidial shapes, and narrower appressoria were seen for in Δ*MaChsV* mutant appressoria (Fig 7D). Finally, Δ*MaChsVII* mutant formed aberrant and longer germ tubes as compared to wild type (Fig 7D, in all cases complemented mutants were similar to wild type). Moreover, the turgor pressure of appressoria from Δ*MaChsV* and Δ*MaChsVII* mutants was significantly reduced (*P* < 0.05) compared to that of wild type (Fig 7E). The relative expression of *Pr1A* in conidia of the Δ*MaChsIII*, Δ*MaChsV*, Δ*MaChsVII* mutants were significantly lower than in wild type (*P* < 0.01). In appressoria, lower *Pr1A* expression in Δ*MaChsVII* mutant and higher *Pr1A* expression in the Δ*MaChsV* mutant were found compared to wild type (*P* < 0.01; Fig 7F).

**Fig 8:**
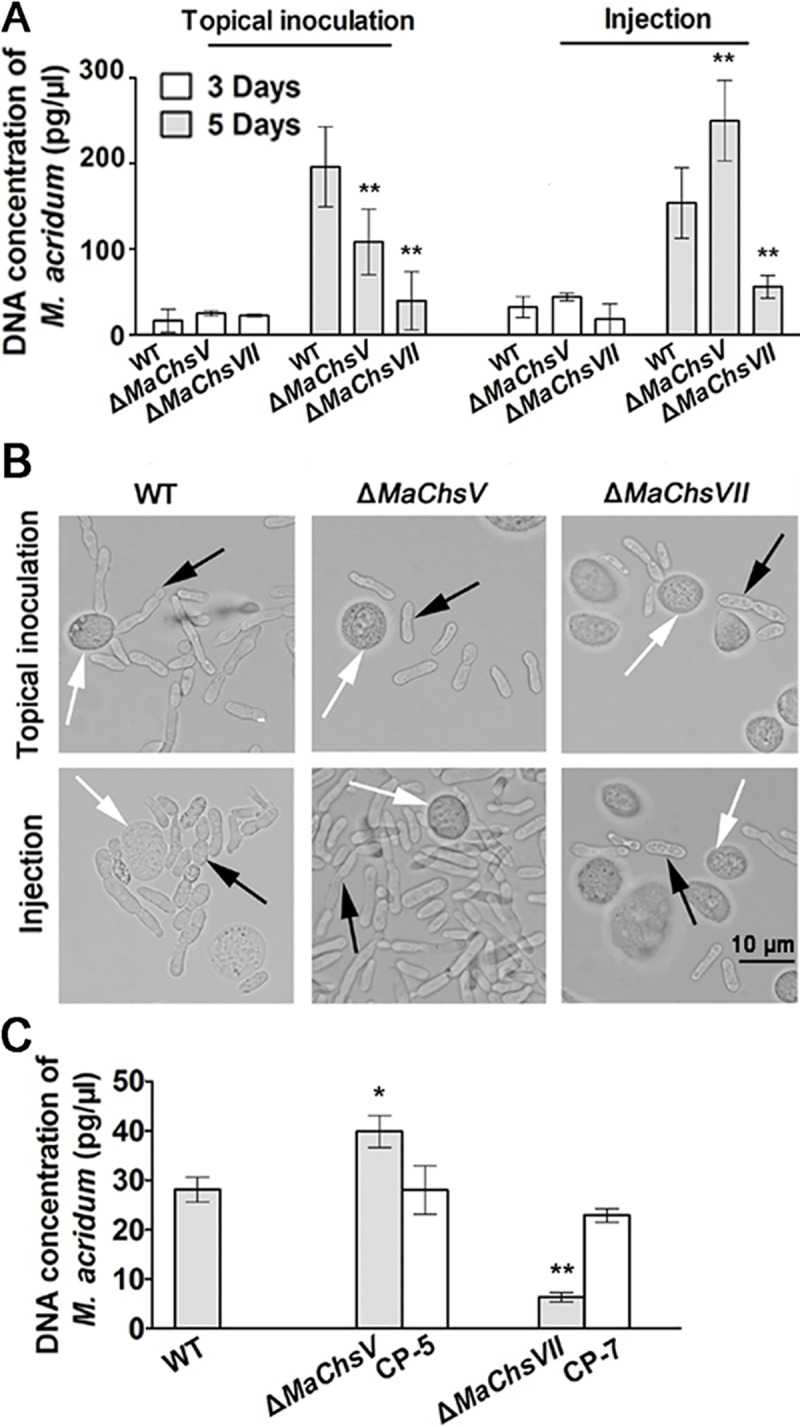
Fungal growth in the insect hemolymph and *in vitro*. (A) Quantification of fungal growth. Total fungal DNA corresponding to the wild type, Δ*MaChsV* and Δ*MaChsVII* mutants in the locust hemolymph after topical inoculation and intrahemocoel injection was determined over the indicated time course as detailed in the Methods section. (B) Representative images of *M*. *acridum* hyphal bodies in the locust hemolymph at 5 d post-treatment. Black arrows indicate hyphal bodies. White arrows indicate locust hemocytes. (C) Quantification of fungal growth in isolated locust hemolymph (*in vitro*). Total DNA concentrations of the wild type, Δ*MaChsV* and Δ*MaChsVII* mutants grown in isolated locust hemolymph *in vitro*. A single asterisk above bars denotes significant difference, *P* < 0.05; double asterisks above bars denote significant difference, *P* < 0.01. Error bars indicate standard errors of three trials.

In order to examine whether any of the chitin synthases affected fungal growth *in insecta*, the production of hyphal bodies during infection was examined. After topical inoculation, no significant differences were found in the concentration of fungal hyphal bodies between the different fungal strains tested 3 d post-inoculation as determined by quantification of fungal DNA (*P* > 0.05; Fig 8A). At 5 d post-inoculation, however, significant reductions in total fungal concentrations were observed for the Δ*MaChsV* or Δ*MaChsVII* mutants as compared to the wild type (*P* < 0.05; Fig 8A and 8B). In intrahemocoel injection assays, treatment with the Δ*MaChsVII* mutant resulted in significantly lower fungal growth 3 and 5 d post-injection as compared to the wild type parent (*P* < 0.05; Fig 8A and 8B), whereas higher total fungal growth was seen for the Δ*MaChsV* mutant 5 d post-injection (*P* < 0.05; Fig 8A and 8B, note complemented mutants were similar to wild type in fungal proliferation in the locust in all experiments tested). In addition, growth of fungal cells cultured in *in vitro* on locust hemolymph without hemocytes was assessed by real time PCR (qPCR). These data indicated that growth of Δ*MaChsV* was significantly faster than that of wild type at 3 d after incubation, while the growth of Δ*MaChsVII* was significantly slower than that of the wild type (*P* < 0.05; Fig 8C). As indicated previously all complemented mutants were essentially identical to the wild type and mutants not indicated were also similar to wild type.

### Deletion of *MaChsIII*, *MaChsV*, or *MaChsVII* reduces the ability of the fungus to block insect immune responses

**Fig 9:**
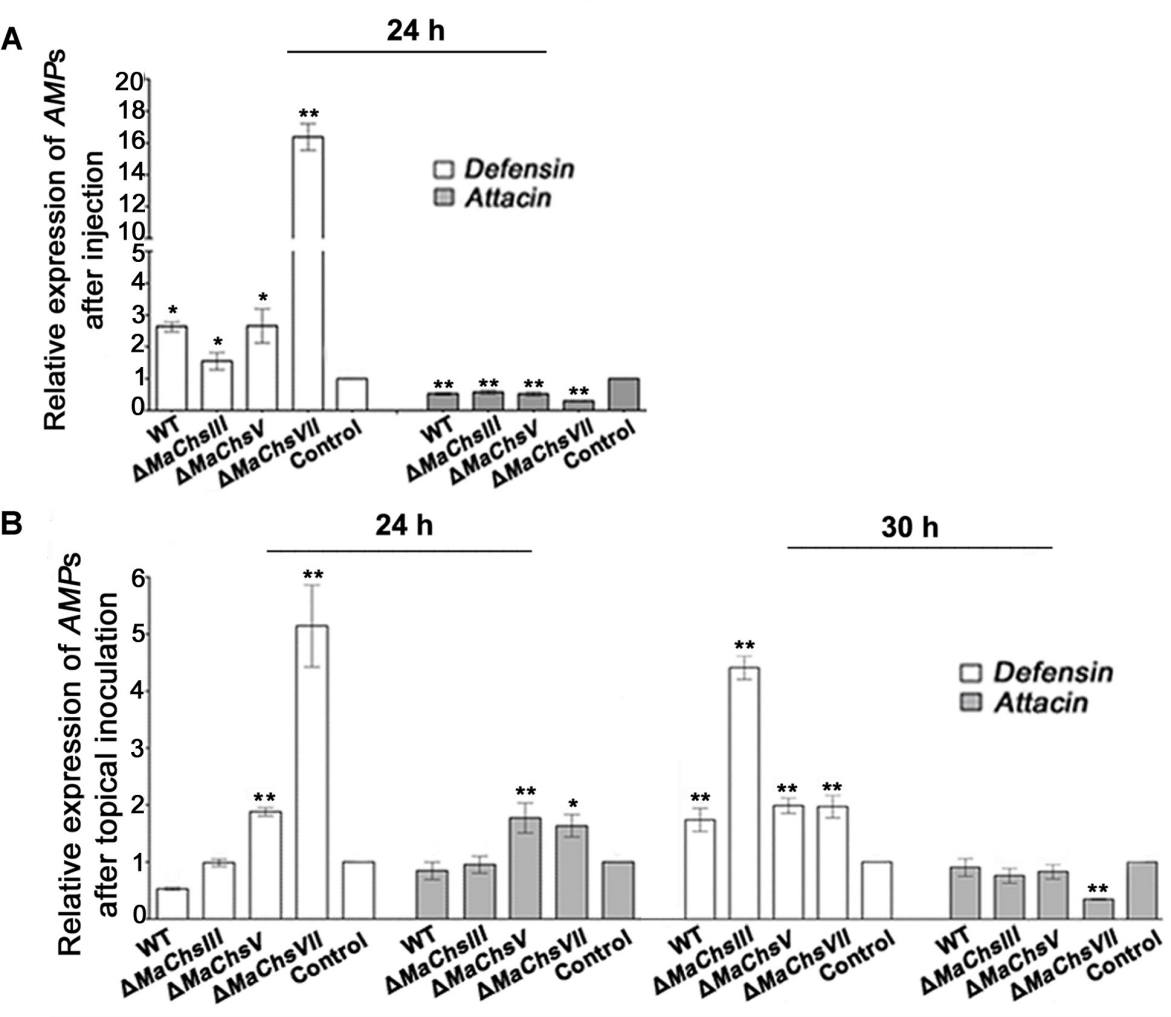
Expression analysis of antimicrobial peptide genes (*AMPs*) in locust. (A) The relative expression levels of *Defensin* and *Attacin* at 24 h after injection. (B) The relative expression levels of *Defensin* and *Attacin* at 24 h and 30 h after topical inoculation. The locusts without treatment were used as the control. A single asterisk above bars denotes significant difference, *P* < 0.05; double asterisks above bars denote significant difference, *P* < 0.01. Error bars indicate standard errors of three trials.

In order to probe whether the *MaChs* genes contributed to the ability of the fungus to evade specific immune defenses three aspects of the host immune reaction were examined; (1) the expression of *Defensin* and *Attacin*, Toll- and/or Imd-activating antimicrobial peptide genes, in the insect fat bodies, (2) nodule formation was quantified after fungal infection, and (3) phenoloxidase (PO) activity was quantified in the insect hemolymph. At 24 h after post-injection of fungal conidia, *Defensin* expression was elevated (~2–3 fold) to similar levels by the wild type and Δ*MaChsIII* and Δ*MaChsV* mutants as compared to mock treated controls, however, *Defensin* levels were induced ~5–6 fold above the wild type levels by the Δ*MaChsVII* mutant (Fig 9A). Under similar conditions, no changes in *Attacin* expression was seen (Fig 9A). In topical bioassays, a slightly different pattern of *Defensin* and *Attacin* expression was seen. At 24 h post-topical inoculation, higher expression of both *Defensin* and *Attacin* were detected in locusts infected with the Δ*MaChsV* and Δ*MaChsVII* mutants compared to control, whereas no significant change was found in those infected by the wild type and Δ*MaChsIII* strains (Fig 9B). At 30 h post-topical inoculation, the expression of *Defensin* in locusts infected by fungal strains was higher than that of control, and with *Defensin* expression in locusts infected with the Δ*MaChsIII* mutant the highest. However, at this time point, no significant changes of the *Attacin* expression was found among the locusts infected by the different fungal strains and control, with exception of a slight but significant (*P* < 0.01), lower expression of the gene in locusts infected by the Δ*MaChsVII* mutant (Fig 9B).

**Fig 10:**
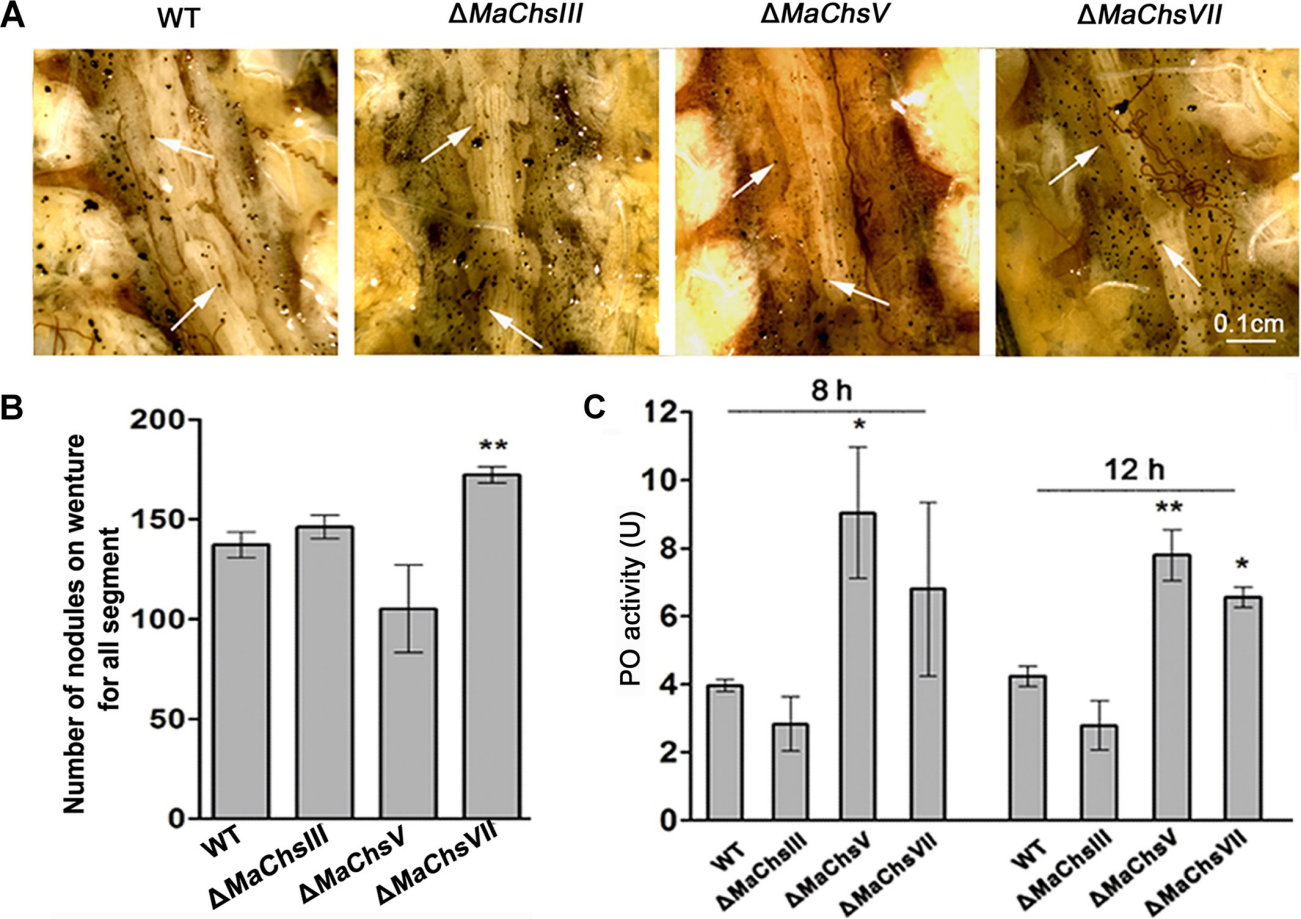
Determination of nodulation and phenoloxidase (PO) activity in locust. (A) The nodule formation in insect inner body walls at 12 h after injection. Arrows indicate typical nodules. (B) The number of nodules after injection at 12 h on insect inner body walls. (C) Phenoloxidase (PO) activity in hemolymph at 8 h and 12 h after inoculation with various strains. A single asterisk above bars denotes significant difference, *P* < 0.05; double asterisks above bars denote significant difference, *P* < 0.01. Error bars indicate standard errors of three trials.

Examination of nodule formation after infection revealed a wide distribution of nodules formed on the inner body walls of the insect after injection of fungal conidia (Fig 10A). Quantification of the number of nodules formed indicated an increase after infection by the Δ*MaChsVII* mutant (173 ± 8 nodules/locust) as compared to the wild type (137 ± 10 nodules/locust) on the ventral diaphragm (*P* < 0.01; Fig 10B). The Δ*MaChsV* showed a slight reduction in nodule formation that was, however, not significant (*P* > 0.05). Quantification of host phenoloxidase (PO) activity revealed elevated PO activity for the Δ*MaChsV* and Δ*MaChsVII* mutants, 8 h post-intrahemocoel injection, however, only the former was significant (*P* < 0.05) at this time point (Fig 10C). By 12 h post-inoculation, PO activity for both mutants (Δ*MaChsV* and Δ*MaChsVII*) were significantly increased (*P* < 0.01 for Δ*MaChsV* and *P* < 0.05 for Δ*MaChsVII*), above wild type. No significant changes in nodulation or PO activity were seen for the Δ*MaChsIII* mutant.

**Fig 11:**
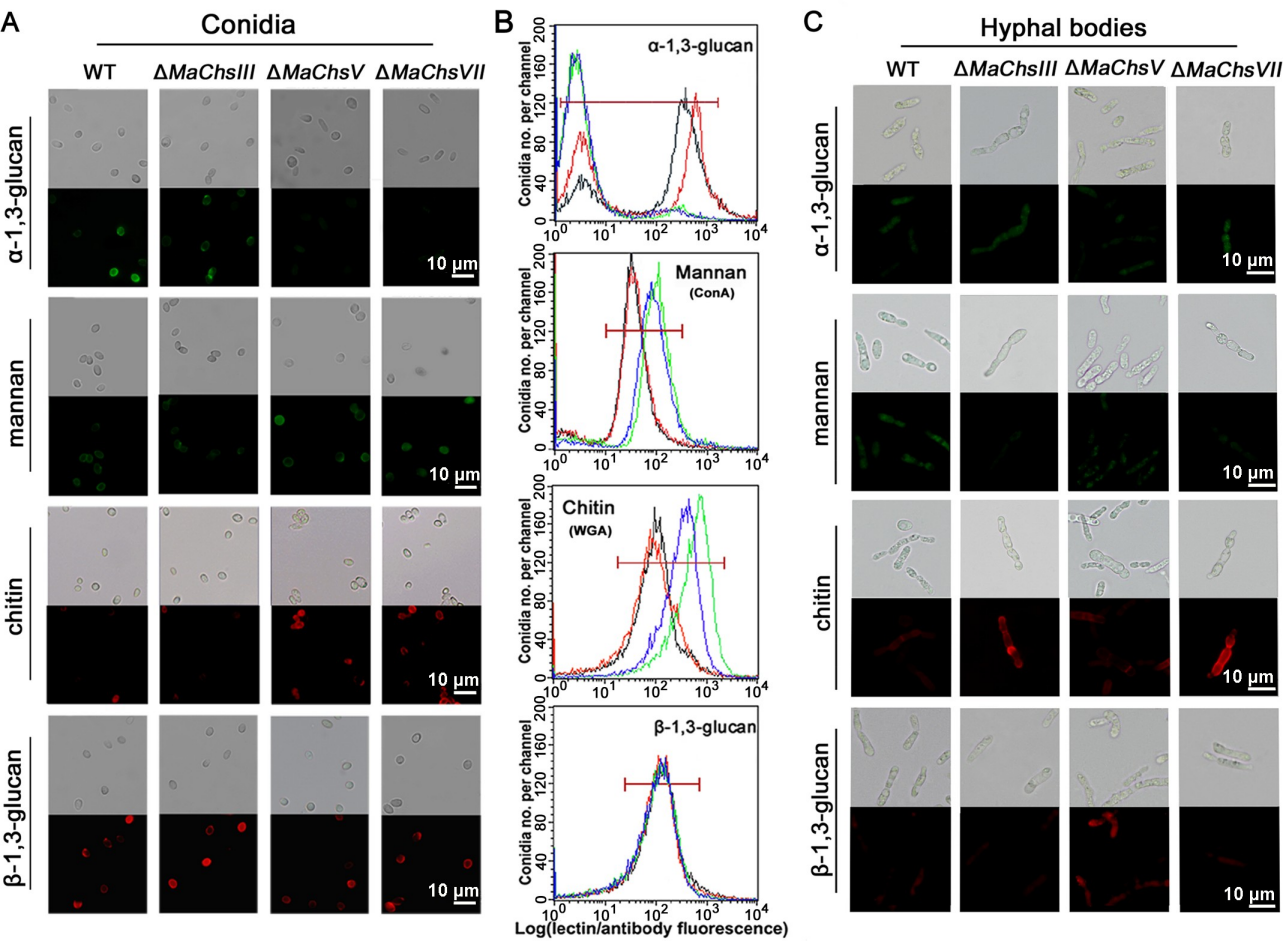
Detection of fungal cell wall surface carbohydrates. (A) Detection of α-1,3-glucan, mannan, chitin, and β-1,3-glucan on conidial surface using fluorescent staining. (B) Representative flow cytometry results for distributions of lectin and antibody binding by *M*. *acridum* conidia from wild type (black line), Δ*MaChsIII* (red line), Δ*MaChsV* (green line), and Δ*MaChsVII* (blue line). (C) Detection of α-1,3-glucan, mannan, chitin, and β-1,3-glucan on hyphal body surface using fluorescent staining. The α-1,3-glucan on fungal cell wall surface staining by α-1,3-glucan-specific antibody, IgM clone MOPC-104E, with Alexa Fluor 488 goat anti-mouse IgG secondary antibody. Mannan on fungal cell wall surface staining by fluorescein-labeled ConA in fungal cell walls. Chitin on fungal cell wall surface staining by fluorescein-labeled WGA. The β-1,3-glucan on fungal cell wall surface staining by β-1,3-glucan-specific antibody with Alexa Fluor 594 goat anti-mouse IgG (H+L) secondary antibody.

In order to determine examine alterations in cell surface carbohydrates that could potentially contribute to the virulence phenotypes of the *MaChs* mutants, the surface carbohydrates of those displaying altered virulence, were examined using a series of fluorescently labeled lectins and antibodies. These included probing using wheat germ agglutinin (WGA), Concanavilin A (ConA), a β-1,3-glucan antibody, and an α-1,3-glucan antibody. Microscopic and flow cytometry analyses revealed that conidia from the Δ*MaChsV* and Δ*MaChsVII* mutants decreased WGA and ConA binding, as well as a slight decrease in staining with the α-1,3-glucan, but no changes in binding of the β-1,3-glucan antibody as compared to conidia derived from the wild-type strain (Fig 11A and 11B). Examination of the surface carbohydrates of the fungal hyphal bodies isolated from infected insects indicated increased WGA reactivity towards Δ*MaChsIII* and Δ*MaChsVII* hyphal bodies, with decreased ConA binding as compared to the wild type (Fig 11C). In addition, hyphal bodies derived from the Δ*MaChsV* mutant showed increased fluorescence intensity when stained with the β-1,3-glucan antibody (Fig 11C).

## Discussion

Chitin, a biopolymer consisting of β-1,4-linked *N*-acetylglucosamine, is an important component of the fungal cell wall and the exoskeletons of insects but is not found in the cellular membranes of plants or animals [42]. Chitin synthases play important roles in fungal cell wall stability, yeast growth, filamentous hyphal development and fungal pathogenicity [17, 43]. To date, chitin synthases have been examined in only a few fungal species and have not been systemically studied in insect pathogenic fungi. In this study, all seven chitin synthase genes identified in the locust-specific entomopathogenic fungus, *M*. *acridum*, were characterized. The *MaChs* mutant phenotypes have been summarized (S2 Table). Results revealed that the seven chitin synthase genes of *M*. *acridum* perform important functions in growth and development, stress tolerances, as well as in cell wall integrity and virulence. All of the *MaChs* genes with the exception of *MaChsVI* were found to contribute to the ability of fungal conidia to germinate. Disruption of *MaChsIII*, *MaChsV*, *MaChsVI* and *MaChsVII* also affected fungal vegetative growth, and all of the *MaChs* genes were found to positively contribute to the production of conidia. All of the *MaChs* genes with the exception of *MaChsI* were involved in fungal stress tolerances. *MaChsIII*, *MaChsV* and *MaChVII* were involved in the fungal cell wall integrity and the ability of conidia to withstand biophysical pressure. *MaChsIII*, *MaChsV* and *MaChVII* also contributed to fungal virulence. Deletion of *MaChsIII*, *MaChsV*, or *MaChsVII* led to defects in fungal appressorium formation and/or impaired the ability of the fungus to undergo a dimorphic transition allowing it to grow in the insect hemolymph. Loss of *MaChsIII*, *MaChsV*, or *MaChsVII* also led to enhanced ability of the insect immune system to recognize and respond to the invading fungus, presumably due to alterations of conidial surface structures including changes in cell wall carbohydrates: This was supported by lectin and antibody binding experiments data that also suggest that β-glucans are exposed in hyphal bodies of Δ*MaChsV*.

The fungal cell wall acts as the barrier protecting the cell from external stress and maintains the shape of the cell [44]. In many fungal species, chitin can contribute more than 50% of the dry weight of the cell wall [45] and is thought to be essential for fungal cell wall integrity and cell wall rigidity. In addition, chitin is found at the septal membranes that separate cells in other filamentous fungal multicellular structures. Consequently, the enzymes responsible for chitin synthesis, i.e. chitin synthases, can be expected to play critical roles in the maintenance of fungal cell wall integrity. However, the observation of multiple chitin synthase genes in all fungi, suggests that some may have specialized roles in cell wall architecture. In *M*. *oryzae*, the *Chs2* (class II) mutant had increased sensitivity to cell wall disrupting agents [6]. In *Botrytis cinerea*, disruption of *Bcchs1* (class II) resulted in cell wall weakening [46]. In *C*. *graminicola*, the class VII *CHS* gene contributes to cell wall integrity of conidia and vegetative hyphae [47]. In this study, conidia of the Δ*MaChsIII*, Δ*MaChsV and* Δ*MaChsVII* mutants became more fragile than those of the wild type. Imaging of the cell wall by TEM showed that Δ*MaChsIII*, Δ*MaChsV* and Δ*MaChsVII* mutants had much thinner cell walls than wild-type cells. In addition, disruption of *MaChsIII*, *MaChsV* and *MaChsVII* led to significant changes in cell wall composition compared with other strains. These results demonstrated that *MaChsIII*, *MaChsV* and *MaChVII* are involved in the fungal cell wall integrity. The thinner cell walls and significant changes of cell wall composition in Δ*MaChsIII*, Δ*MaChsV* and Δ*MaChsVII* mutants may result in the cell wall become more fragile.

Disruption of cell wall biosynthetic genes or treatments with cell wall perturbing agents often results in compensatory alterations in the cell wall, such as enhancing the synthesis of the cell wall polymers, in an attempt to maintain cellular integrity [2]. Here, we measured the levels of the major carbohydrate constituents of the cell walls of mutants of the *Chs* genes. Levels of β-1,3-glucan in the Δ*MaChsIII*, Δ*MaChsV*, and Δ*MaChsVII* mutants and chitin levels in the Δ*MaChsV* and Δ*MaChsVII* mutants significantly higher than those of the wild-type strain. It is possible that the cell wall integrity defects in the Δ*MaChsIII*, Δ*MaChsV*, and Δ*MaChsVII* mutants resulted in compensatory effects in which the cell increased cell wall constituents in an attempt to maintain cellular membrane integrity. Fungal cells containing higher chitin levels are usually less resistant to cell wall disturbing agents such as calcofluor white, whereas cells with lower chitin levels are more resistant to cell wall disturbing agents [48, 49]. In *Aspergillus fumigatus*, the Δ*ChsA*/*C*/*B*/*G* and Δ*ChsG* mutants were able to withstand high concentrations calcofluor white or Congo red, becoming hyper-resistant to cell wall disturbing agents [18]. In this study, increased fungal cell wall chitin levels seen in the Δ*MaChsV* and Δ*MaChsVII* mutants resulted in greater sensitivity to calcofluor white than the wild type parent.

Conidial yield, stress tolerances, and virulence are very important parameters for entomopathogenic fungi with respect to biological control applications of the fungus [50–52]. Previous works in other filamentous fungi have shown that chitin synthesis impacts fungal conidiation. In *A*. *nidulans*, *ChsA* (class I), *ChsC* (class III) and *ChsD* (class IV) are involved in the regulation of conidiation [14, 53, 54]. In *A*. *fumigatus*, the quadruple Δ*ChsA*/*C*/*B*/*G* deletion mutant formed abnormal vesicles and showed a drastic reduction in conidiation. The two MMD (myosin motor domain)-containing chitin synthase genes *CsmA* (*ChsV*) and *CsmB* (*ChsVII)* also play important roles in conidiogenesis [18]. In *Magnaporthe oryzae*, *Chs1* (class III) mutant produced pear-shaped, single-celled conidia, while other *Chs* mutants formed three-celled pyriform conidia with normal morphology. The conidiation in *Chs2* (class II) and *Chs6* (class V) mutants were decreased over 5-fold compared with their parental strain [6]. In this study, similar results were found in that all the *MaChs* mutants exhibited defects in conidiation, especially the Δ*MaChsIII*, Δ*MaChsIV*, Δ*MaChsV* and Δ*MaChsVII* mutants displayed significant reductions in conidial yield (~60%). This observation is consistent with the *MaChsIII*, *MaChsIV* and *MaChsVII* showing higher expression levels than the other *MaChs* genes during the conidia formation stage. As potential pest biological control agent, tolerances to abiotic stress including heat and UV-B irradiation are critical for entomopathogenic fungi to survive in various environments [28, 55]. Our data showed that *MaChs* genes with the exception of *MaChsI* are involved in fungal stress tolerances. The Δ*MaChsIII*, Δ*MaChsV*, Δ*MaChsVI*, and Δ*MaChsVII* mutants displayed significantly increased sensitivity to heat shock, and the Δ*MaChsIII* and Δ*MaChsVII* mutants were more sensitive to UV-B radiation. In addition, the Δ*MaChsII* and Δ*MaChsIV* mutants showed decreased sensitivity to heat shock and UV-B radiation, respectively.

Changes in the chitin content in the cell wall of various fungi have been shown to result in defects in appressorium formation and hyphal growth, as well as increased susceptibility to host immune defenses [6, 9, 18, 20]. In *M*. *oryzae*, the *Chs1* (class III) and *Chs7* (class VI) genes play important roles in appressorium formation. The Δ*Chs1* (class III) and Δ*Chs7* (class VI) mutants were reduced in virulence [6]. The *Chs6* (class V) of *M*. *oryzae* plays critical roles in penetration and development invasive hyphae in plant cells, and deletion of *Chs6*, resulted in avirulence [6]. Our data show that disruption of the *MaChsI*, *MaChsII*, *MaChsIV*, or *MaChsVI* did not affect fungal virulence, whereas inactivation of *MaChsIII* or *MaChsV* significantly reduced fungal virulence and deletion of *MaChsVII* essentially resulted in loss of pathogenicity. The Δ*MaChsIII*, Δ*MaChsV*, and Δ*MaChsVII* mutants showed defects in appressorium formation, structures which play a critical role in the initiation of fungal infection of the insect [56]. The appressorium morphology of the Δ*MaChsV* and Δ*MaChsVII* mutants was aberrant, likely impacting the ability of these structures to penetrate the insect cuticle. This observation is consistent with the observation that the *MaChsIII*, *MaChsV*, and *MaChsVII* genes were more highly expressed in the appressorium formation stage as compared to the other *MaChs* genes. The appressoria of fungal pathogen need very rigid cell walls to control the high osmotic pressure in the stage of infection [43]. In this study, the Δ*MaChsV* and Δ*MaChsVII* mutants exhibited lower appressorial turgor pressure as compared to the wild type. Previous research has shown that weakening of the cell wall can lead to reduced fungal virulence [4, 57]. Here, deletion of the *MaChsIII*, *MaChsV*, or *MaChsVII* genes resulted in fragility of the cell wall of *M*. *acridum*. After reaching the insect hemocoel, the penetrating hyphae undergo a dimorphic transition producing hyphal bodies that spread and grow within the insect [29, 30]. Hyphal body growth of the Δ*MaChsVII* mutant was reduced, consistent with its decreased virulence in both topical and intrahemocoel injection assays. In contrast, the hyphal body growth for the Δ*MaChsV* mutant was increased in the insect hemocoel. Intriguingly, for this mutant, virulence was decreased in topical bioassays, but was slightly higher than the wild type in injection assays. One explanation for this observation is that disruption of *MaChsV* reduced the formation and/or turgor pressure of appressoria, resulting in impaired ability of the fungus to penetrate the cuticle. Thus, this mutant, appears to have a specific deficiency in penetration (as seen for several other genes, *e*.*g*. a bifunctional catalase-peroxidase [50]), but once the cuticle is bypassed can grow quickly (as indicated by the hyphal body proliferation data).

Humoral immunity is considered the dominant immune response of insects [58], and includes the Toll and Imd signal pathways [59]. *Attacin* and *Defensin* are antimicrobial peptides present in locusts that are regulated by the Imd and Toll pathways [60–63]. Our data indicate that when compared to wild type, the Δ*MaChsV* and Δ*MaChsVII* mutants were unable to block certain aspects of the insect humoral immune response. After topical inoculation, higher expression levels of both *Attacin* and *Defensin* in locusts infected by the Δ*MaChsV* and Δ*MaChsVII* mutants suggest activation of Imd and Toll pathways, that can at least partially account for the decreased virulence seen for these mutants. In injection assays, only *Defensin* appeared up-regulated in response to the Δ*MaChsVII* mutant. Not too surprisingly, these data suggest that the mode of infection can affect the ability of the Toll/Imd pathways to recognize and/or respond to the fungal pathogen. It should be borne in mind that topical application represents that “natural” route of infection and would represent the likely ability of the host to respond to the pathogen. In addition, an increase in the number of nodules was seen in infections with the Δ*MaChsVII* mutant indicated that the insect cellular immune response was also active and hence provides additional explanation for the reduced virulence seen for this mutant. The systemic immune response is triggered not only by the cell wall compounds, but also by the enzymatic activity of fungal proteases [34–36]. The expression levels of the *Pr1A* protease gene in conidia and appressoria revealed lower expression in the chitin synthase mutants as compared to the wild type, with exception of appressoria derived from the Δ*MaChsV* mutant.

On the cell wall, fungal-specific components are ideal targets recognized by the host innate immune system [64, 65]. Deletion of the *MaChsIII*, *MaChsV*, or *MaChsVII* genes resulted in altered cell surface carbohydrates potentially leading to increased detection in the insect’s immune system. Cell wall components, including hydrophobins and collagen-like protein, can shield the fungus from host recognition [66–68]. Conidial surface hydrophobicity was decreased in the Δ*MaChsIII*, Δ*MaChsV*, and Δ*MaChsVII* mutants. Since some cell wall components are fungal-genera specific [64], they are ideal targets for recognition by the host innate immune system. In this respect, most mannans are recognized by many membrane-bound receptors that include the mannose receptor, DC-SIGN or SIGN-R1, galectin 3, Dectin-2, TLR4, and/or Mincle [69–71]. Cell wall mannan exposure in conidia from the Δ*MaChsV* and Δ*MaChsVII* mutants was increased, while mannan exposure was decreased in hyphal bodies of these mutants. The presence of α-1,3-glucan is known to mask certain fungal pathogens from host immune recognition [72]. Fluorescence detection and flow cytometry data showed that the conidia of Δ*MaChsV* and Δ*MaChsVII* mutants had decreased α-1,3-glucan, while no obvious differences of this polymer were found among the hyphal bodies from all fungal strains. Thus, although the ability to detect the original infectious cells (conidia) may remain, the hyphal bodies produced appear to be still capable of shielding themselves (at least partly). β-1,3-glucan is recognized by insect host immune system and can activate the host Toll pathway [34]. In this work, the increased β-1,3-glucan antibody binding, and hence presumably increased β-1,3-glucan exposure were seen in hyphal bodies derived from the Δ*MaChsV* mutant (no significant changes were found on conidia from all fungal strains). This result is puzzling, as this strain displays increased virulence in injection assays and increased hyphal body proliferation. At present we cannot account for this observation, with the possibility that other compensating factors may be able to overcome any potential negative consequences of the potential for increased immune activation by these cells. Chitin can also stimulate different host immune pathways [2, 73, 74]. An increase in chitin exposure was seen for conidia of the Δ*MaChsV* and Δ*MaChsVII* mutants and in hyphal bodies of the Δ*MaChsIII* and Δ*MaChsVII* mutants, consistent (but not necessarily accounting for) the decreased virulence of these mutants. Overall, the changes of surface carbohydrates in conidia and hyphal bodies of the Δ*MaChsIII*, Δ*MaChsV* and Δ*MaChsVII* mutants may lead to the enhanced insect immune responses, with the increased virulence of the Δ*MaChsV* mutant in injection assays, the outlier finding. Alterations in surface carbohydrates in hyphal bodies as opposed to conidia has been previously observed [29, 30], with our data indicating that chitin synthases contribute to this process.

In summary, members of chitin synthase family in *M*. *acridum*, a model entomopathogenic fungus, were systematically characterized. As a specialized insect pathogen, *M*. *acridum* possess distinct mechanisms for infection of their locust hosts [60, 75]. Our results indicate that different chitin synthase genes of *M*. *acridum* play diverse roles in growth, stress tolerances, as well as in cell wall integrity and virulence. *MaChsIII*, *MaChsV*, and *MaChsVII*, were found to contribute to fungal virulence. Disruption of *MaChsIII*, *MaChsV* and *MaChsVII* leads to defects in fungal appressorium formation and blocks the ability of the fungus to inhibit insect immune recognition/activation to fungus that could in part be due to increased exposure of recognition signals on the surface of conidia and/or hyphal bodies. These data provide new insights into the multifunctional roles of chitin synthases in fungal development and virulence.

## Materials and methods

### Microbial strains and culturing

*M*. *acridum* CQMa102 (China General Microbiological Culture Collection Center, CGMCC, No. 0877), and all constructed strains were routinely cultured in 1/4 SDAY (1% dextrose, 0.25% mycological peptone, 0.5% yeast extract and 2% agar, w/v) plates for 3–15 days at 28°C for conidial production [76], as well as on potato dextrose agar (PDA) (Solarbio, Beijing, China) or Czapek-dox agar (CZA, 3% sucrose, 0.3% NaNO_3_, 0.1% K_2_HPO_4_, 0.05% KCl, 0.05% MgSO_4_ and 0.001% FeSO_4_ plus 1.5% agar) as needed. *Escherichia coli* DH5α and *Agrobacterium tumefaciens* AGL-1 were used for DNA manipulations and transformations and routinely grown in Luria-Bertani (LB) broth.

### Phylogenetic analysis

Accession numbers for the seven *M*. *acridum* chitin synthases are as follows; MaChsI (XP_007814354.1), MaChsII (XP_007814507.1), MaChsIII (XP_007810158.1), MaChsIV (XP_007813853.1), MaChsV (XP_007814979.1), MaChsVI (XP_007814966.1) and MaChsVII (XP_007814978.1). The BLAST program was used to select the fungal Chs protein on the NCBI website (www.ncbi.nlm.nih.gov). Phylogenetic dendrograms were constructed for the Chs protein sequences retrieved from the genomes of *M*. *oryzae* (Mo), *Candida albicans* (Ca), *S*. *cerevisiae* (Sc), *U*. *maydis* (Um), *A*. *fumigatus* (Af) and *M*. *acridum* (Ma) by neighbor-joining method using MEGA (ver. 7.0) (http://www.megasoftware.net) with a bootstrap test of 1,000 replicates.

### Construction of the targeted gene disruption complementation strains

Construction of targeted gene disruption vectors for each of the 7 *M*. *acridum Chs* genes followed a similar plan. Briefly, 5′ and 3′ flanking sequences of each gene were cloned into pK2-PB [77] to create pK2-PB-MaChs-5′-*bar*-3′ vectors (the *bar* gene flanked by the cloned sequences). The 5′ and 3′ flanking sequences of each *MaChs* gene was amplified from *M*. *acridum* genomic DNA by PCR using primer pairs listed in S1 Table. Restriction cloning sites and primers were used for PCR verification as given for each gene (S1 Table). Final vectors were confirmed by sequencing and the gene disruption vector was mobilized into *A*. *tumefaciens* AGL-1 for transformation in *M*. *acridum*. Fungal genomic DNA was prepared using Fungal DNA Kit (Omega, Beijing, China).

Complementation vectors for each gene were constructed using the pK2-sur platform conferring resistance to chlorimuron ethyl [78]. For each *Chs* gene, the entire ORF (1.5–2.5 kb) as well as promoter (~2 kb) and terminator (~1 kb) sequences were amplified by PCR using *M*. *acridum* genomic DNA as the template and primer pairs as given (S1 Table). The resulting PCR products were digested with *Eco*RV/*Bam*HI, and inserted into the pK2-sur. Vectors were checked by sequencing and the resultant plasmids were transformed into *A*. *tumefaciens* AGL-1 for transformation into each respective *Chs* mutant strain for complementation.

Fungal transformation using *A*. *tumefaciens* was performed as described previously [79]. The *MaChs* disruption mutants were initially selected CZA supplemented with 500 μg ml^-1^ glufosinate ammonium resistance. Initial screens of putative *MaChs*-disruption transformants were performed using PCR using primer pairs MaChs-VF/Pt-R2 and MaChs-VR/Bar-F2 as listed for each gene in S1 Table. Complemented transformants were initially selected on CZA containing 20 μg ml^-1^ chlorimuron ethyl and confirmed by PCR using the primer pair of Sur-F/Sur-R (S1 Table). Southern blotting was used to verify the integrity of select disruption and complemented mutant strains.

### qRT-PCR analysis

The expression levels of the *MaChs* genes were analyzed by qRT-PCR. Total RNA was isolated from *M*. *acridum* conidia, hyphae, appressoria and hyphal bodies. Conidia were harvested from 1/4 SDAY plates after 15 d of growth. Hyphae were harvested from 1/4 SDAY plates after 18 h of growth. Appressoria were collected from wings of *Locusta migratoria manilensis* at 8 h, 20 h and 30 h post-inculation. Briefly, 5 locust wings were immersed in 2 ml of 1×10^8^ conidia ml^-1^ conidial suspension for 10 min and rinsed with sterile water, and the entire sample used for total RNA extraction. Hyphal bodies were collected from infected insect hemolymph at 5 d after topical inoculation as described [77]. Total RNA was extracted using an RNeasy Mini Kit (QIAGEN, Hilden, Germany). cDNA was synthesized from 1 μg of DNaseI-treated total RNA with oligo-dT primer in 20 μl PrimeScript RT Master Mix (TaKaRa, Dalian, China). Real-time PCR was performed using the SYBR-Green PCR Master Mix kit (Bio-Rad, USA) in the iCycler PCR System (Bio-Rad, USA). The relative transcript levels of each gene were quantified using the 2^−ΔΔC^_T_ method [80]. The *M*. *acridum gpdh* (GenBank accession number: EFY84384), a gene encoding glyceraldehyde 3-phosphate dehydrogenase, was used as an internal control. All PCR amplifications were conducted in triplicate and the entire experiment repeated with a biological duplicate sample.

### Phenotypic assays

Fungal growth assays examining stress sensitivity were performed as follows: conidia from 15 d 1/4 SDAY plates were harvested and suspended in doubled-distilled H_2_O and final concentration of conidial suspension was adjusted to 1×10^6^ conidia ml^-1^. Aliquots of conidial suspensions (2 μl, 1×10^6^ conidia ml^-1^) were spotted with a micropipette onto the center of various plates including 1/4 SDAY amended with Congo red (CR; 500 μg ml^-1^), calcofluor white (CFW; 50 μg ml^-1^), H_2_O_2_ (6 mmol l^-1^), SDS (0.1 g l^-1^), sorbitol (1 mol l^-1^) and NaCl (0.5 mol l^-1^). Plates were incubated at 28 ^o^C for 5–7 d and colony diameters quantified [77]. Three replicate plates were used for each condition/experiment and the entire experiment repeated with 1–2 independent batches of conidia.

Conidial yield was estimated on fungal colonies grown in 24-well microtiter plates. Each well contained 1 ml 1/4 SDAY and was inoculated with 2 μl conidial suspension (1×10^6^ conidia ml^-1^) and incubated for 15 d at 28 ^o^C. Conidia were harvested from wells by flooding with 3 ml water containing 0.5% Tween 20 and the subsequent cells in the suspension quantified by counting using haemocytometer. For conidial germination assays an aliquot of 100 μl of 1×10^7^ conidia ml^-1^ was spread on 1/4 SDAY and incubated at 28 ^o^C. Conidial germination was examined microscopically every 2.5 h after staining with CFW buffer [81]. Cells were photographed using a fluorescence microscope (Olympus BX51, Tokyo, Japan). Three replicate wells were used for each fungal strain and the entire experiment repeated using a separate batch of fungal conidia. Fungal hyphal elongation rates were measured using an inverted microscope (Nikon Ti-E, Tokyo, Japan). Images were captured at 2 h intervals from 10 h to 18 h after inoculation, and analyzed with the NIS-Elements BR3.2 software.

Fungal tolerances to heat shock and ultraviolet radiation (UV-B) were tested as described [82]. Briefly, a 100-μl aliquot of conidial suspension at a concentration of 5×10^6^ conidia ml^-1^ was transferred to sterile eppendorf tubes and immediately placed in a water bath at 45 ^o^C for 2, 4, 6 and 8 h. After exposure, a 20-μl aliquot was spread evenly on PDA agar. Plates were incubated at 28 ^o^C for 24 h. With respect to ultraviolet radiation (UV-B) tolerances, fungal cells were spread evenly onto 1/4 SDAY plates. The plates were immediately exposed to irradiances of 1350 mW m^-2^ for 2, 4, 6 and 8 h, respectively. After irradiation, the plates were incubated in darkness at 28 ^o^C for 24 h. The germination rates were calculated via microscopic observation.

Samples were analyzed by transmission electron microscopy (TEM) as described [83]. Briefly, conidia were collected from 1/4 SDAY plates 28°C for 15 days and washed three times with phosphate buffer solution (PBS, pH 7.4). Following centrifugation, conidia were fixed with 4% glutaraldehyde at 4°C overnight. Fixed samples were washed with 0.1 M PBS buffer three times and fixed with 2% osmium tetroxide in 0.1 M PBS for 2 h at room temperature, followed by dehydration in the gradients of 50–90% ethanol and 100% acetone. Samples were embedded in resin and ultrathin sectioned. Sections were stained with uranyl acetate and lead citrate and observed on the TEM (Hitachi H-7500, Tokyo, Japan). From TEM images, the cell wall thickness was measured from 3 to 5 ultrathin sections of conidia using the Nis-elements BR3.2 software (Nikon).

### Measurement of the cell wall contents, integrity and hydrophobicity

Fungal strains were grown in 50 ml 1/4 SDY at 28 ^o^C for 2 days before harvesting of the growing hyphae by centrifugation (6,000 × g, 3 min). Cell were washed three times with 30 ml of 2% SDS and total chitin content was determined by acid hydrolysis of the fungal cell wall as described [84]. Total β-1,3-glucan content was determined by degradation of the alkali-insoluble fraction of the cell wall in reaction mixtures containing 1 mg ml^-1^ zymolyase 100T as described previously [83]. Mannoproteins were extracted with 1 M NaOH at 100°C from cell walls and measured using Folin’s reagent and bovine serum albumin as the standard [85]. All experiments were repeated three times with independent batches of growing cells.

To test the fungal cell wall integrity, 500 μl of freshly harvested conidial suspensions (1×10^7^ conidia ml^-1^) in 1.5 ml eppendorf tubes were centrifuged at 12,000 × g for 5 min. After centrifugation, the concentration (C_1_) of intact conidia was determined using a hemocytometer. Conidia with normal shape were considered as intact conidia. The fragility was calculated using the following equation: (1×10^7^–C_1_)/1×10^7^.

Conidial hydrophobicity was measured using the microbial adhesion to hydrocarbons assay as described with slight modifications [86]. Briefly, conidia were harvested from 1/4 SDAY after 15 d and washed into reaction buffer (0.2 g MgSO_4_, 1.8 g urea, 7.26 g KH_2_PO_4_, 22.2 g K_2_PO_4_ per L, pH 7.1). Conidial suspensions were adjusted to 1×10^7^ conidia ml^-1^ and 3 ml dispensed into 5 ml tubes. To each tube, 300 μl hexadecane was added, the sample was mixed thoroughly on a vortex mixer (three times for 30 s) and then allowed to equilibrate at room temperature for 15 min after which the organic (hexadecane) layer removed. To remove any residual hexadecane, the tubes were cooled to 4°C and any solidified hexadecane remaining removed. The concentration (C_2_) of conidial in the aqueous phase was determined by counting. The hydrophobic index was calculated using the following equation: (1×10^7 _^C_2_)/1×10^7^.

### Fluorescence detection of fungal cell wall surface carbohydrates and flow cytometry

Fungal conidia were harvested from 1/4 SDAY after 15 d of growth. Hyphal bodies were collected from the locust hemolymph 4 d post-injection with 5 μl of conidial suspension (1×10^7^ conidia ml^-1^) as described [30]. All the samples were washed 3 times with 0.01 M PBS buffer and immunofluorescent labeling and detection of α-1,3-glucan and β-1,3-glucan in fungal cell walls were performed using IgM clone MOPC-104E and Alexa Fluor 488 goat antimouse IgM (Invitrogen), and β-1,3-glucan using β-1,3-glucan-specific antibody, Alexa Fluor 594 goat antimouse IgG anti-body (Invitrogen) as described previously [30]. Fungal surface carbohydrates containing β-1,4 N-acetylglucosamine or mannose were visualized using conjugated wheat germ agglutinin (WGA) Texas Red @-X conjugates (Vector Laboratories, Burlingame, CA, USA) or fluorescein-labeled Concanavalin A (ConA) (Vector Laboratories, Burlingame, CA, USA), respectively, and as described previously [30].

Fluorescent signals of conidia were quantified by BD FACSCalibur with an argon laser, with the excitation wavelength set at 488 nm (Ex: 488 nm) and the emitted light detector at 530 nm (Em: 530±15 nm), adjusted to a fixed channel using standard Brite Beads prior to determining fluorescence of α-1,3-glucan and mannose. And with the excitation wavelength set at 488 nm (Ex: 488 nm) and the emitted light detector at 630 nm (Em: 630±15 nm) to quantify fluorescence of β-1,3-glucan and chitin. Samples were diluted to 4×10^4^ conidia ml^-1^, and briefly mixed on a vortex mixer before introduction to sheath fluid. Data acquisition and manipulation were performed with BD CellQuest Pro and FACS Express v3, and fluorescence was measured for 24,000 conidia. Experiments were performed on at least three independent batches of conidia.

### Locust immune response assays

The expression levels of the locust *Attacin* (GenBank accession number: AB757753) and *Defensin* (GenBank accession number: KU516094) antimicrobial peptide gene, were analyzed by qRT-PCR. Total RNA was isolated from dissected *L*. *migratoria manilensis* fat bodies after topical infection with *M*. *acridum* (1×10^8^ conidia ml^-1^) after 24 and 30 h, or injection with *M*. *acridum* (1×10^7^ conidia ml^-1^) after 24 h post-treatment. Total RNA extraction, cDNA synthesis, and qRT-PCR were conducted as described earlier. The *L*. *migratoria manilensis β*-*actin* gene (GenBank accession number: KC118986) was used as an internal control. All experiments were performed in triplicate.

To determine the phenoloxidase (PO) activity in the locust hemolymph, samples was harvested from *L*. *migratoria manilensis* after topical infection with *M*. *acridum* (1×10^8^ conidia ml^-1^) 8 and 12 h post-inoculation by cutting off of the proleg and collecting hemolymph droplets on ice. Immediately after collection 100 μl of hemolymph was added to 1 ml PBS buffer (50 mM, pH 6.5). The mixture was then centrifuged at 3,099 × g for 10 min at 4°C in order to remove cells and debris. PO activity was measured using a TriStar multimode microplate reader LB941 (Berthold, Bad Wildbad, Germany). Protein quantification was performed using the Folin-Phenol Protein Quantification Kit (Dingguo, Beijing, China). One unit of PO activity was defined as ΔA_490_ = 0.001 after 60 minutes, similar to previously described [87, 88].

The nodules transformation was performed with 30 *L*. *migratoria manilensis* fifth-instar nymphs after 12 h which injected with 5 μl aqueous suspension containing 1×10 ^8^ conidia ml^-1^ into the hemocoel as described previously [89]. A mid-dorsal cut was made along the full length of the body. The gut and fat bodies were removed to expose the inner ventral surface and nodules were counted routinely in all abdominal segments under a dissecting microscope. The number of nodules was calculated as previously described with some modifications [90]. Nodule size was factored in as follow; single nodules were tabulated for sizes ranging from 50 to 90 μm, with nodules > 100 μm considered as two nodules. All experiments were repeated three times.

### Insect bioassays

Fifth-instar nymphs of *L*. *migratoria manilensis* (Meyen) were used for bioassays as described previously [77]. Conidial germination and appressorium formation were examined on locust hind wings using a previously described method [91]. Appressorial turgor pressure was assayed using a previously described method [92]. For the fungal growth rates in insect hemolymph *in vivo*, conidial suspensions from different fungal strains were injected (5 μl of 2×10^6^ conidia ml^-1^ aqueous conidial suspensions) into the locust hemocoel cavity through the third abdominal segment or topically inoculated (3 μl of 2×10^7^ conidia ml^-1^ paraffin oil conidial suspensions) onto pronotums of locusts. Treated locusts were reared at 28°C with a 16:8 h (light–dark) photoperiod and bled at day 3 and day 5 after inoculation. Three cohorts of 10 treated locusts were bled (30 μl blood per locust) for genomic DNA extraction. Hyphal bodies *in vivo* were observed and photographed under a microscope by bleeding infected locusts 5 d post inoculation. For the fungal growth rates in insect haemolymph *in vitro*, 10 μl of conidial suspension (1×10^6^ conidia ml^-1^) was inoculated into a 2 ml microcentrifuge tube which containing 500 μl fresh locust hemolymph, from which host cells were removed by centrifugation at 30 × g for 10 min at 4°C. Samples were stand at 28°C on a rotary shaker at 250 rpm for 3 days. Genomic DNA from samples was extracted at 48 h or 24 h intervals as described above. The concentration of fungal genomic DNA was examined by qPCR using primer pair of ITS-F/ITS-R (S1 Table) to determine the fungal growth rate.

### Statistical analysis

All datasets were analyzed with SPSS 16.0 program (IBM, Armonk, NY, USA). The mean 50% lethality time (LT_50_) and mean 50% inhibition time (IT_50_) were estimated using the Data Processing System program [93]. Shapiro-Wilk test and Levene's test were used for testing the normality and homogeneity of variances, respectively. When the data distributed normally, one-way analysis of variance (ANOVA) followed by Tukey’s test was selected, or t tests was selected. Tukey’s honestly significant difference test was used to separate means at α = 0.05 or 0.01. All experiments were repeated at least three times.

## Supporting information

S1 Table Primers used in this study. (DOCX)

S2 Table Overview of *MaChs* mutant phenotypes. (DOCX)

S1 Fig Phylogenetic analysis of Chs family proteins. Phylogenetic dendrograms were constructed for the Chs protein sequences retrieved from the genomes of *M*. *oryzae* (Mo), *Candida albicans* (Ca), *S*. *cerevisiae* (Sc), *U*. *maydis* (Um), *A*. *fumigatus* (Af) and *M*. *acridum* (Ma) by neighbor-joining method using MEGA (ver. 7.0) (http://www.megasoftware.net) with a bootstrap test of 1,000 replicates. The bar indicates 0.2 distance units.
(TIF)

S2 Fig Disruption and complementation of *MaChsI* in *M. acridum*. (A) Schematic illustration of the *MaChsI* disruption in *M*. *acridum*. The probe was obtained by PCR using primers ChsI-PF and ChsI-PR. (B) Design of the *MaChsI* complementation plasmid. (C) Southern blot analysis of the transformants hybridized by the probe. About 10 μg genomic DNA of WT, Δ*MaChsI*, CP-1 was digested with *Pst*I. WT: the wild type; Δ*MaChsI*: *MaChsI*-disruption transformant; CP-1: *MaChsI*-complementary transformant. (TIF)
(TIF)

S3 Fig Disruption and complementation of *MaChsII* in *M. acridum*. (A) Schematic illustration of the *MaChsII* disruption in *M*. *acridum*. The probe was obtained by PCR using primers ChsII-PF and ChsII-PR. (B) Design of the *MaChsII* complementation plasmid. (C) Southern blot analysis of the transformants hybridized by the probe. About 10 μg genomic DNA of WT, Δ*MaChsII*, CP-2 was digested with *Eco*RI. WT: the wild type; Δ*MaChsII*: *MaChsII*-disruption transformant; CP-2: *MaChsII*-complementary transformant.
(TIF)

S4 Fig Disruption and complementation of *MaChsIII* in *M. acridum*. (A) Schematic illustration of the *MaChsIII* disruption in *M*. *acridum*. The probe was obtained by PCR using primers ChsIII-PF and ChsIII-PR. (B) Design of the *MaChsIII* complementation plasmid. (C) Southern blot analysis of the transformants hybridized by the probe. About 10 μg genomic DNA of WT, Δ*MaChsIII*, CP-3 was digested with *Eco*RV and *Xho*I. WT: the wild type; Δ*MaChsIII*: *MaChsIII*-disruption transformant; CP-3: *MaChsIII*-complementary transformant.
(TIF)

S5 Fig Disruption and complementation of *MaChsIV* in *M. acridum*. (A) Schematic illustration of the *MaChsIV* disruption in *M*. *acridum*. The probe was obtained by PCR using primers ChsIV-PF and ChsIV-PR. (B) Design of the *MaChsIV* complementation plasmid. (C) Southern blot analysis of the transformants hybridized by the probe. About 10 μg genomic DNA of WT, Δ*MaChsIV*, CP-4 was digested with *Nco*I. WT: the wild type; Δ*MaChsIV*: *MaChsIV*-disruption transformant; CP-4: *MaChsIV*-complementary transformant.
(TIF)

S6 Fig Disruption and complementation of *MaChsV* in *M. acridum*. (A) Schematic illustration of the *MaChsV* disruption in *M*. *acridum*. The probe was obtained by PCR using primers ChsV-PF and ChsV-PR. (B) Design of the *MaChsV* complementation plasmid. (C) Southern blot analysis of the transformants hybridized by the probe. About 10 μg genomic DNA of WT, Δ*MaChsV*, CP-5 was digested with *Spe*I and *Eco*RI. WT: the wild type; Δ*MaChsV*: *MaChsV*-disruption transformant; CP-5: *MaChsV*-complementary transformant.
(TIF)

S7 Fig Disruption and complementation of *MaChsVI* in *M. acridum*. (A) Schematic illustration of the *MaChsVI* disruption in *M*. *acridum*. The probe was obtained by PCR using primers ChsVI-PF and ChsVI-PR. (B) Design of the *MaChsVI* complementation plasmid. (C) Southern blot analysis of the transformants hybridized by the probe. About 10 μg genomic DNA of WT, Δ*MaChsVI*, CP-6 was digested with *Apa*I and *Xho*I. WT: the wild type; Δ*MaChsVI*: *MaChsVI*-disruption transformant; CP-6: *MaChsVI*-complementary transformant.
(TIF)

S8 Fig Disruption and complementation of *MaChsVII* in *M. acridum*. (A) Schematic illustration of the *MaChsVII* disruption in *M*. *acridum*. The probe was obtained by PCR using primers ChsVII-PF and ChsVII-PR. (B) Design of the *MaChsVII* complementation plasmid. (C) Southern blot analysis of the transformants hybridized by the probe. About 10 μg genomic DNA of WT, Δ*MaChsVII*, CP-7 was digested with *Apa*LI and *Xho*I. WT: the wild type; Δ*MaChsVII*: *MaChsVII*-disruption transformant; CP-7: *MaChsVII*-complementary transformant.
(TIF)

S9 Fig Colony morphology of *M. acridum* wild type, Δ*MaChsI*, Δ*MaChsII*, Δ*MaChsIV* and Δ*MaChsVI* mutants. Colony morphology of the *MaChs* deletion mutants on 1/4 SDAY or 1/4 SDAY supplemented with 0.1% SDS, 1.5 mol l^-1^ Sorbitol, 0.5 mol l^-1^ NaCl, 500 μg ml^-1^ CR (Congo red), 50 μg ml^-1^ CFW (calcofluor white), 6 mmol l^-1^ H_2_O_2_ at 28°C. The fungal colonies were photographed after 5 d of incubation. Bar scale = 0.5 cm.
(TIF)

S10 Fig Fungal dry weight of *M. acridum* wild type and Δ*MaChs* mutant hyphae in liquid 1/4 SDY at 28°C for 3 days by shaking at 200 rpm. A single asterisk above bars denotes significant difference, *P* < 0.05. Error bars indicate standard errors of three trials.
(TIF)

S11 Fig Germination rates of the various *M. acridum* strains at 7.5 h after incubation on 1/4 SDAY. (TIF)

S12 Fig Mannoproteins in *M. acridum* wild type and *MaChs* mutant cells. A single asterisk above bars denotes significant difference, *P* < 0.05; double asterisks above bars denote significant difference, *P* < 0.01. Error bars indicate standard errors of three trials.
(TIF)

S13 Fig Insect bioassays. (A) Survival of locusts after topical inoculation with 5 μl Tween-80 (0.05%) containing 1×10^8^ conidia ml^-1^ of wild type and Δ*MaChsI*, Δ*MaChsII*, Δ*MaChsIV*, Δ*MaChsVI* mutants. (B) Survival of locusts after injection with 5 μl sterile water containing 1×10^6^ conidia m^-1^ of wild type and Δ*MaChsI*, Δ*MaChsII*, Δ*MaChsIV*, Δ*MaChsVI* mutants. Error bars indicate standard errors of three trials.
(TIF)

S14 Fig The hydrophobicity of conidia from wild type, Δ*MaChsI*, Δ*MaChsII*, Δ*MaChsIV*, Δ*MaChsVI* mutants. ns indicates no significant difference, *P* > 0.05. Error bars indicate standard errors of three trials.
(TIF)
