# Supplementary material for: Members of chitin synthase family in Metarhizium acridum differentially affect fungal growth, stress tolerances, cell wall integrity and virulence
Source: PLoS Pathog. 2019 Aug 28;15(8):e1007964. doi: 10.1371/journal.ppat.1007964 (PMC6713334; doi:10.1371/journal.ppat.1007964)
Supplement: S1 Table — (DOCX) [file ppat.1007964.s001.docx]

S1 Table Primers used in this study.

| Primers | Sequences (5′-3′) | Remarks | Amplification efficiencies |
| --- | --- | --- | --- |
| MaChsI-LF | GACGGCCAGTGCCAAGCTTGCCTAACCTCGCTCAAT | Used to clone the 5′ end of *MaChsI* |  |
| MaChsI-LR | CGGATCCCTCGAGTCTAGCGTGGGAAGTCAACAACA |  |  |
| MaChsI-RF | GCTGGCCGCCCATGGGATTCTTGGCTCCGCCGACTT | Used to clone the 3′ end of *MaChsI* |  |
| MaChsI-RR | ATGACATGATTACGAATTATTTCAGCACCGTCCACC |  |  |
| MaChsI-VF | GGTTGAGTTCGCTGAGGC | Used for screening the *MaChsI*-disruption transformants |  |
| MaChsI-VR | CGATTCAGAACGGCTACA |  |  |
| MaChsI-PF | AATGCGTTTCTGGTGTACTGA | Used to clone the probe of *MaChsI* |  |
| MaChsI-PR | TGAGCGAGGTTAGGCATTCT |  |  |
| MaChsI-FF | CGGGATCCATGGTGAGCAAGGGCGAGGAGAGAAGCCTCATCTCAAGTCT | Used to clone the DNA fragment encompassing the *MaChsI* open reading frame |  |
| MaChsI-FR | GCGATATCTTATCATTACTTGTACAGCTCGTCAACTCCTCTAAACATTCGAA |  |  |
| MaChsII-LF | GACGGCCAGTGCCAAGCTGGTTTCCCGCAACACTCT | Used to clone the 5′ end of *MaChsII* |  |
| MaChsII-LR | CGGATCCCTCGAGTCTAGATTGACGCCACGAGCACA |  |  |
| MaChsII-RF | GCTGGCCGCCCATGGGATAGGTGCTTGTGGCGAGATTA | Used to clone the 3′ end of *MaChsII* |  |
| MaChsII-RR | ATGACATGATTACGAATTTCTCCGATTCCCTTATTGCT |  |  |
| MaChsII-VF | GTCATACGGAGTGAAGGTG | Used for screening the *MaChsII*-disruption transformants |  |
| MaChsII-VR | ACGCTGCTAACCTTCTCG |  |  |
| MaChsII-PF | AGGTGCTTGTGGCGAGATTA | Used to clone the probe of *MaChsII* |  |
| MaChsI-PR | TTTGGTGTTTGTGAAGAGGG |  |  |
| MaChsII-FF | CGGGATCCATGGTGAGCAAGGGCGAGGAGGGGGCTGCTTATCAGTTTCC | Used to clone the DNA fragment encompassing the *MaChsII* open reading frame |  |
| MaChsII-FR | GCGATATCTTATCATTACTTGTACAGCTCGTCACTCTTCAGGCTGCTTCCAA |  |  |
| MaChsIII-LF | GACGGCCAGTGCCAAGCTTTGGCTTGGGTGGGTGTA | Used to clone the 5′ end of *MaChsIII* |  |
| MaChsIII-LR | CGGATCCCTCGAGTCTAGGCTCAGCGCAGCGTCATA |  |  |
| MaChsIII-RF | GCTGGCCGCCCATGGGATAGGGTGCTGCCGAGTTTA | Used to clone the 3′ end of *MaChsIII* |  |
| MaChsIII-RR | ATGACATGATTACGAATTTACGAACAGACGCCGATT |  |  |
| MaChsIII-VF | TCCCTGGCACATTCATTC | Used for screening the *MaChsIII*-disruption transformants |  |
| MaChsIII-VR | CCACCGTATTCCCTCCTT |  |  |
| MaChsIII-PF | GGTTCTCGCTTGCTTCCT | Used to clone the probe of *MaChsIII* |  |
| MaChsIII-PR | GCCTTGTCTGAGCCCTTG |  |  |
| MaChsIII-FF | CGGGATCCATGGTGAGCAAGGGCGAGGAGATTGAGGCGATTGAGGAAA | Used to clone the DNA fragment encompassing the *MaChsIII* open reading frame |  |
| MaChsIII-FR | GCGATATCTTATCATTACTTGTACAGCTCGTCTCTGCGAGCGAAGCAGCAC |  |  |
| MaChsIV-LF | GACGGCCAGTGCCAAGCTAGGGCAGAAATCGGGAGC | Used to clone the 5′ end of *MaChsIV* |  |
| MaChsIV-LR | CGGATCCCTCGAGTCTAGGACGGTATCAACGAATTGTAGC |  |  |
| MaChsIV-RF | GCTGGCCGCCCATGGGATTCATCAAGGGCAAGGGTG | Used to clone the 3′ end of *MaChsIV* |  |
| MaChsIV-RR | ATGACATGATTACGAATTGCGGAGGTGAGTGGATAATAGA |  |  |
| MaChsIV-VF | GTGCGTTCAATGTTCTGC | Used for screening the *MaChsIV*-disruption transformants |  |
| MaChsIV-VR | CCAGACGACATAGGACCAC |  |  |
| MaChsIV-PF | TCATCAAGGGCAAGGGTG | Used to clone the probe of *MaChsIV* |  |
| MaChsIV-PR | TTCATAGTAGTCGGGTGAGATA |  |  |
| MaChsIV-FF | CGGGATCCATGGTGAGCAAGGGCGAGGAGGCAACAGGGGTTATTATTA | Used to clone the DNA fragment encompassing the *MaChsIV* open reading frame |  |
| MaChsIV-FR | GCGATATCTTATCATTACTTGTACAGCTCGTCTGCGTCTGAGTAGTAGCC |  |  |
| MaChsV-LF | GACGGCCAGTGCCAAGCTGACGTGCTATCGAATTGTG | Used to clone the 5′ end of *MaChsV* |  |
| MaChsV-LR | CGGATCCCTCGAGTCTAGCTGGACTAACACGAGCAAA |  |  |
| MaChsV-RF | GCTGGCCGCCCATGGGATCGAGGTGGGTTCTGCTAC | Used to clone the 3′ end of *MaChsV* |  |
| MaChsV-RR | ATGACATGATTACGAATTGGATAAGGAAGTCGGGAATA |  |  |
| MaChsV-VF | TTTGTTGTCCGGTGTAATT | Used for screening the *MaChsV*-disruption transformants |  |
| MaChsV-VR | CACGACGATAAAGAAAGCAG |  |  |
| MaChsV-PF | AAGGGAGGCAATAAAGGTG | Used to clone the probe of *MaChsV* |  |
| MaChsV-PR | ATGATGGCAAGGCGAAGC |  |  |
| MaChsV-FF | CGGGATCCATGGTGAGCAAGGGCGAGGAGGTTCTGATTCATATCCGGTGTCT | Used to clone the DNA fragment encompassing the *MaChsV* open reading frame |  |
| MaChsV-FR | GCGATATCTTATCATTACTTGTACAGCTCGTCCTTACCGCTATTAATATAGGCTC |  |  |
| MaChsVI-LF | GACGGCCAGTGCCAAGCTCGTTCTAAACTCAGCACCAC | Used to clone the 5′ end of *MaChsVI* |  |
| MaChsVI-LR | CGGATCCCTCGAGTCTAGTAAGGGCATAGCGACACC |  |  |
| MaChsVI-RF | GCTGGCCGCCCATGGGATAAAGGAGGCAAGGACAAT | Used to clone the 3′ end of *MaChsVI* |  |
| MaChsVI-RR | ATGACATGATTACGAATTTCCAGTCGCCTGAATACC |  |  |
| MaChsVI-VF | AATCAATCCCAGAAGGTG | Used for screening the *MaChsVI*-disruption transformants |  |
| MaChsVI-VR | CATCAATCACCCAGGTATG |  |  |
| MaChsVI-PF | GCACAACTGGCGAGATAG | Used to clone the probe of *MaChsVI* |  |
| MaChsVI-PR | CCGAAGCAAAACAAAAGC |  |  |
| MaChsVI-FF | CGGGATCCATGGTGAGCAAGGGCGAGGAGGACAAAGAAAGGCAACCGAC | Used to clone the DNA fragment encompassing the *MaChsVI* open reading frame |  |
| MaChsVI-FR | GCGATATCTTATCATTACTTGTACAGCTCGTCAAAAGCAATTCCCACGTCAT |  |  |
| MaChsVII-LF | GACGGCCAGTGCCAAGCTGCTCTTGGTGGCTCTGGATA | Used to clone the 5′ end of *MaChsVII* |  |
| MaChsVII-LR | CGGATCCCTCGAGTCTAGAGTAATCAACGGGCACAA |  |  |
| MaChsVII-RF | GCTGGCCGCCCATGGGATTTTGGGTCGGGTTTGTCT | Used to clone the 3′ end of *MaChsVII* |  |
| MaChsVII-RR | ATGACATGATTACGAATTTGAAGGGTAGTGCGGGAG |  |  |
| MaChsVII-VF | GTAAACAAGGCTAACTGGACC | Used for screening the *MaChsVII*-disruption transformants |  |
| MaChsVII-VR | GAAGATTGGCGGACAGCA |  |  |
| MaChsVII-PF | GCTGCTGGTTCTGACATC | Used to clone the probe of *MaChsVII* |  |
| MaChsVII-PR | AAGGAAAATAACAATACTCTGG |  |  |
| MaChsVII-FF | GACGGCCAGTGCCAAGCTGCTCTTGGTGGCTCTGGATA | Used to clone the DNA fragment encompassing the *MaChsVII* open reading frame |  |
| MaChsVII-FR | TTCTTCTGTCGACGGATCCGAGTTGCCCATTTTGACC |  |  |
| Bar-F2 | GCTCTACACCCACCTGCT | Used for screening the *MaChs*-disruption transformants |  |
| Pt-R2 | CAGCCAAGCCCAAAAAGTG |  |  |
| Sur-F | CGGAATTCGTCGACGTGCCAACGCCACAGTG | Used to clone the *sur* gene |  |
| Sur-R | GCGATATCGTCGACGTGAGAGCATGCAATTCC |  |  |
| MaChsI-QF | ATTACTACCGAGGTGTAC | Used to analyze the expression level of *MaChsI* gene | 106.3% |
| MaChsI-QR | CCATAACACAATCGTCAT |  |  |
| MaChsII-QF | GCTGTTTCCGAAATCTAT | Used to analyze the expression level of *MaChsII* gene | 94.1% |
| MaChsII-QR | TTGACATTCATCCGTATC |  |  |
| MaChsIII-QF | AATTACCAGCGACAACTT | Used to analyze the expression level of *MaChsIII* gene | 106.2% |
| MaChsIII-QR | ACAGGAAGCCAATGAATC |  |  |
| MaChsIV-QF | CTGATCTACCTGGTTTCC | Used to analyze the expression level of *MaChsIV* gene | 101.2% |
| MaChsIV-QR | TTCATGGTGATCTTGCTA |  |  |
| MaChsV-QF | AATTCTTCAACCCTGATG | Used to analyze the expression level of *MaChV* gene | 100.3% |
| MaChsV-QR | TTAATATAGGCTCTCTTCG |  |  |
| MaChsVI-QF | AGGAATATCAAGGGTGTA | Used to analyze the expression level of *MaChVI* gene | 97.7% |
| MaChsVI-QR | TGAGCTATGGCGTATTTC |  |  |
| MaChsVII-QF | GATGACGCAGCTATTGTG | Used to analyze the expression level of *MaChsVII* gene | 94.8% |
| MaChsVII-QR | TCAATCTGTCGGTCCATA |  |  |
| Pr1A-QF | TCCTCTCTTCACTCCTCAG | Used to analyze the expression level of *Pr1A* | 109.3% |
| Pr1A-QR | ATACGGGCAATACCATCCT |  |  |
| Gpdh-QF | AGATGGAGGAGTTGGTGTTG | Used to analyze the expression level of *Gapdh* | 108.2% |
| Gpdh-QR | GACTGCCCGCATTGAGAAG |  |  |
| ITS-F | TGGCATCTTCTGAGTGGTG | Used to determine the concentration of fungal genomic DNA of hyphal bodies | 103.6% |
| ITS-R | CCCGTTGCGAGTGAGTTA |  |  |
| Defensin-L | GCGTCTGTCTCCTCTG | Used to analyze the expression level of *Defensin* | 92.4% |
| Defensin-R | CCCTTGTAGCCCTTGTT |  |  |
| Attacin-L | GTGCTCCTCGTCGTTCTGA | Used to analyze the expression level of *Attacin* | 90.7% |
| Attacin-R | CCCACGCCTTTCTCTCTGT |  |  |
| β-actin-F | CGAAACCTTTAATACCCCAG | Used to analyze the expression level of *β-actin* | 102.5% |
| β-actin-R | CCATCACCAGAATCCAACAC |  |  |
