# Supplementary material for: Members of chitin synthase family in Metarhizium acridum differentially affect fungal growth, stress tolerances, cell wall integrity and virulence
Source: PLoS Pathog. 2019 Aug 28;15(8):e1007964. doi: 10.1371/journal.ppat.1007964 (PMC6713334; doi:10.1371/journal.ppat.1007964)
Supplement: S2 Table — (DOCX) [file ppat.1007964.s002.docx]

S2 Table. Overview of *MaChs* mutant phenotypes*.­

| **Phenotypes** | | | **Δ*MaChsI*** | **Δ*MaChsII*** | **Δ*MaChsIII*** | **Δ*MaChsIV*** | **Δ*MaChsV*** | **Δ*MaChsVI*** | **Δ*MaChsVII*** |
| --- | --- | --- | --- | --- | --- | --- | --- | --- | --- |
| Germination | | | Decrease | Increase | Decrease | Decrease | Increase | No change | Decrease |
| Conidial yield | | | Decrease | Decrease | Decrease | Decrease | Decrease | Decrease | Decrease |
| Stress tolerances | UV-B tolerance | | No change | No change | Decrease | Increase | No change | No change | Decrease |
|  | Thermo tolerance | | No change | Increase | Decrease | No change | Decrease | Decrease | Decrease |
| Virulence | Survival of locusts (topical inoculation) | | No change | No change | Increase  (+) | No change | Increase  (+) | No change | Increase (+++) |
|  | Survival of locusts (injection) | | No change | No change | No change | No change | Decrease | No change | Increase |
|  | Invasion | Conidial surface hydrophobicity | No change | No change | Decrease | No change | Decrease | No change | Decrease |
|  |  | Appressorium formination | ND | ND | Decrease | ND | Decrease | ND | Decrease |
|  | Post-penetration | Proliferation in insect hemolymph | ND | ND | No change | ND | Increase | ND | Decrease |
|  |  | Evasion of insect humoral immune reponses | ND | ND | Impairment | ND | Impairment | ND | Impairment |
|  |  | Evasion of nodulation | ND | ND | No change | ND | No change | ND | Impairment |
| * "ND" indicates not determined. The importance of each Chs in fungal virulence is reflected by the number of "+". | | | | | | | | | |
