# Supplementary figures and images for: Members of chitin synthase family in Metarhizium acridum differentially affect fungal growth, stress tolerances, cell wall integrity and virulence
Source: PLoS Pathog. 2019 Aug 28;15(8):e1007964. doi: 10.1371/journal.ppat.1007964 (PMC6713334; doi:10.1371/journal.ppat.1007964)

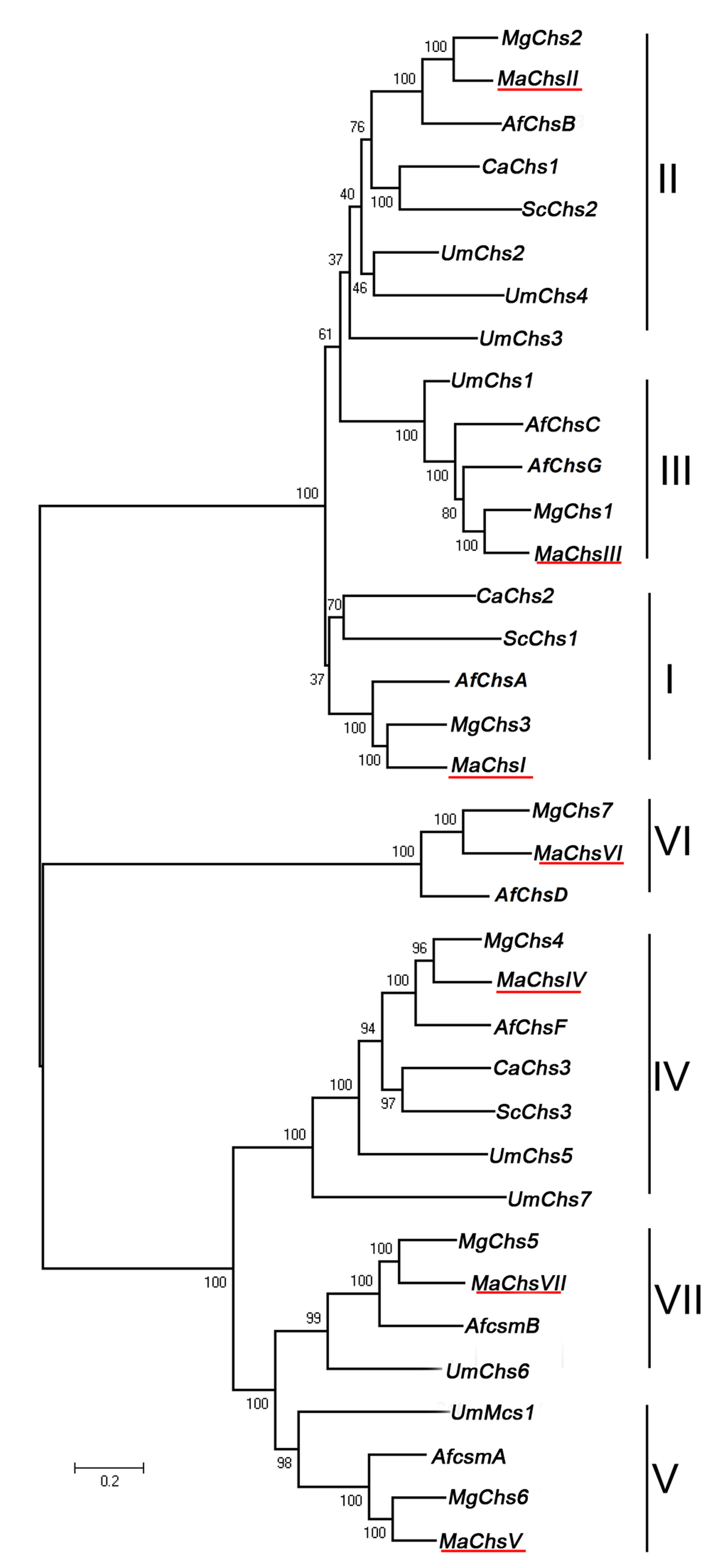

Supplement: S1 Fig — Phylogenetic dendrograms were constructed for the Chs protein sequences retrieved from the genomes of M. oryzae (Mo), Candida albicans (Ca), S. cerevisiae (Sc), U. maydis (Um), A. fumigatus (Af) and M. acridum (Ma) by neighbor-joining method using MEGA (ver. 7.0) (http://www.megasoftware.net) with a bootstrap test of 1,000 replicates. The bar indicates 0.2 distance units. (TIF) [file ppat.1007964.s003.tif]

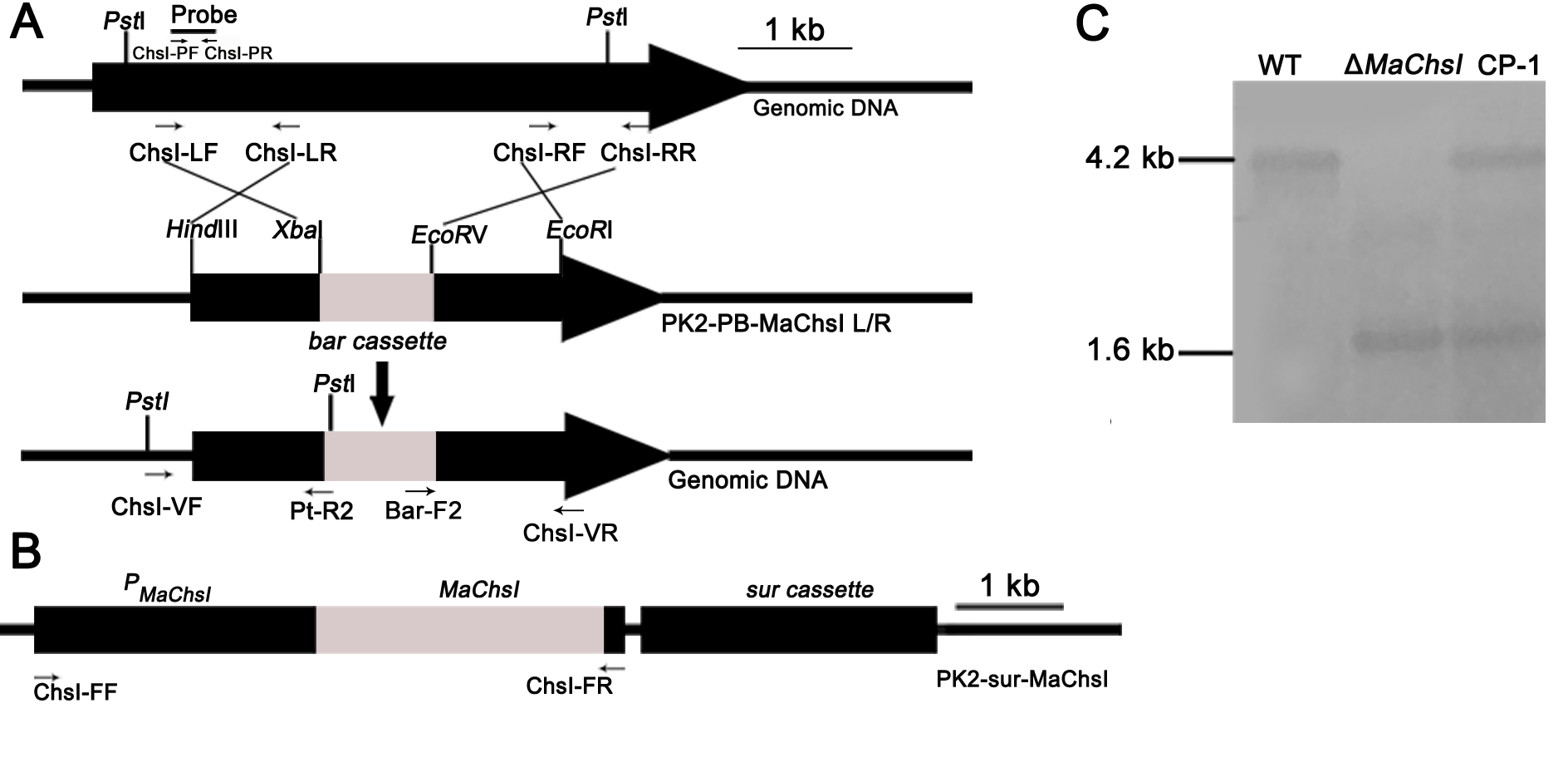

Supplement: S2 Fig — (A) Schematic illustration of the MaChsI disruption in M. acridum. The probe was obtained by PCR using primers ChsI-PF and ChsI-PR. (B) Design of the MaChsI complementation plasmid. (C) Southern blot analysis of the transformants hybridized by the probe. About 10 μg genomic DNA of WT, ΔMaChsI, CP-1 was digested with PstI. WT: the wild type; ΔMaChsI: MaChsI-disruption transformant; CP-1: MaChsI-complementary transformant. (TIF) (TIF) [file ppat.1007964.s004.tif]

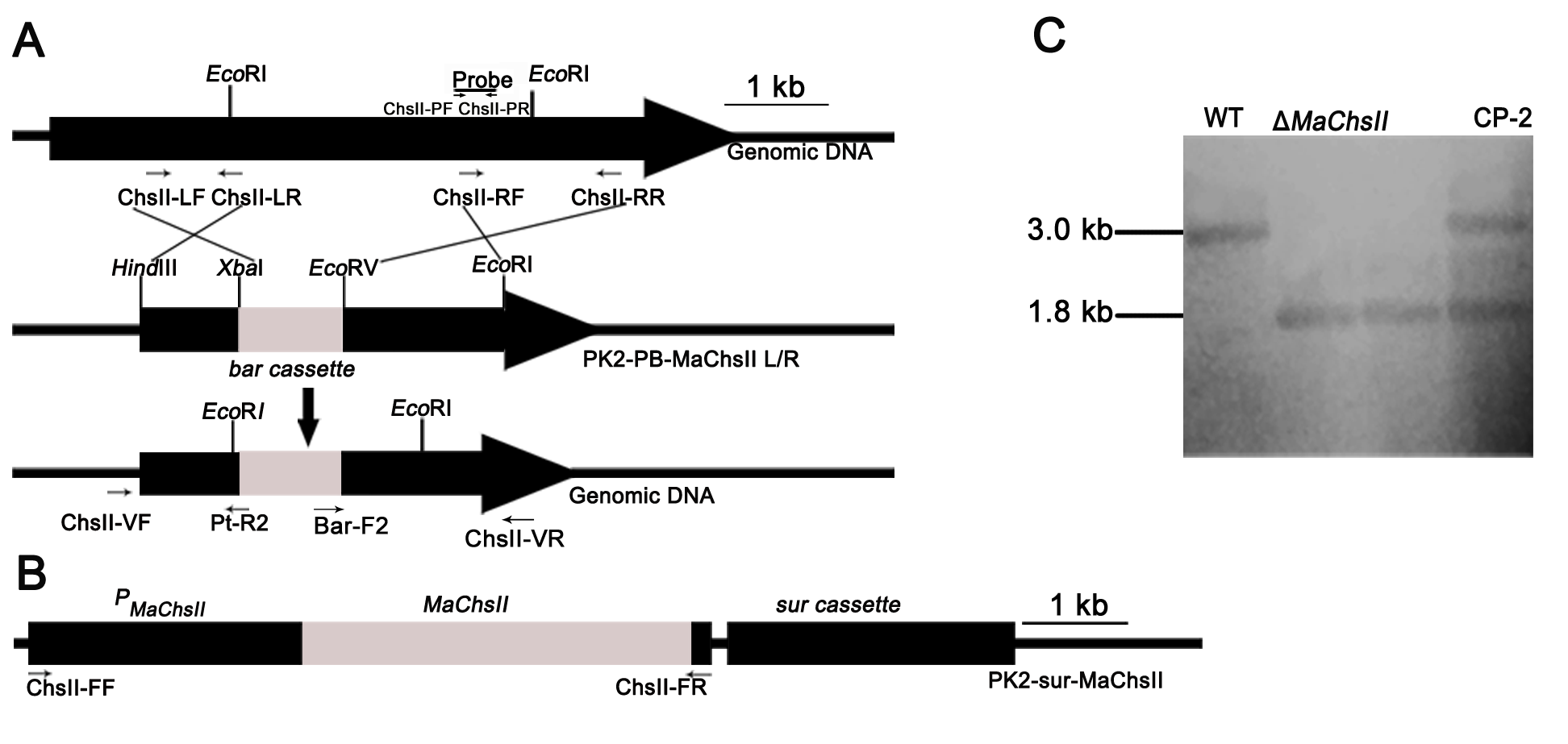

Supplement: S3 Fig — (A) Schematic illustration of the MaChsII disruption in M. acridum. The probe was obtained by PCR using primers ChsII-PF and ChsII-PR. (B) Design of the MaChsII complementation plasmid. (C) Southern blot analysis of the transformants hybridized by the probe. About 10 μg genomic DNA of WT, ΔMaChsII, CP-2 was digested with EcoRI. WT: the wild type; ΔMaChsII: MaChsII-disruption transformant; CP-2: MaChsII-complementary transformant. (TIF) [file ppat.1007964.s005.tif]

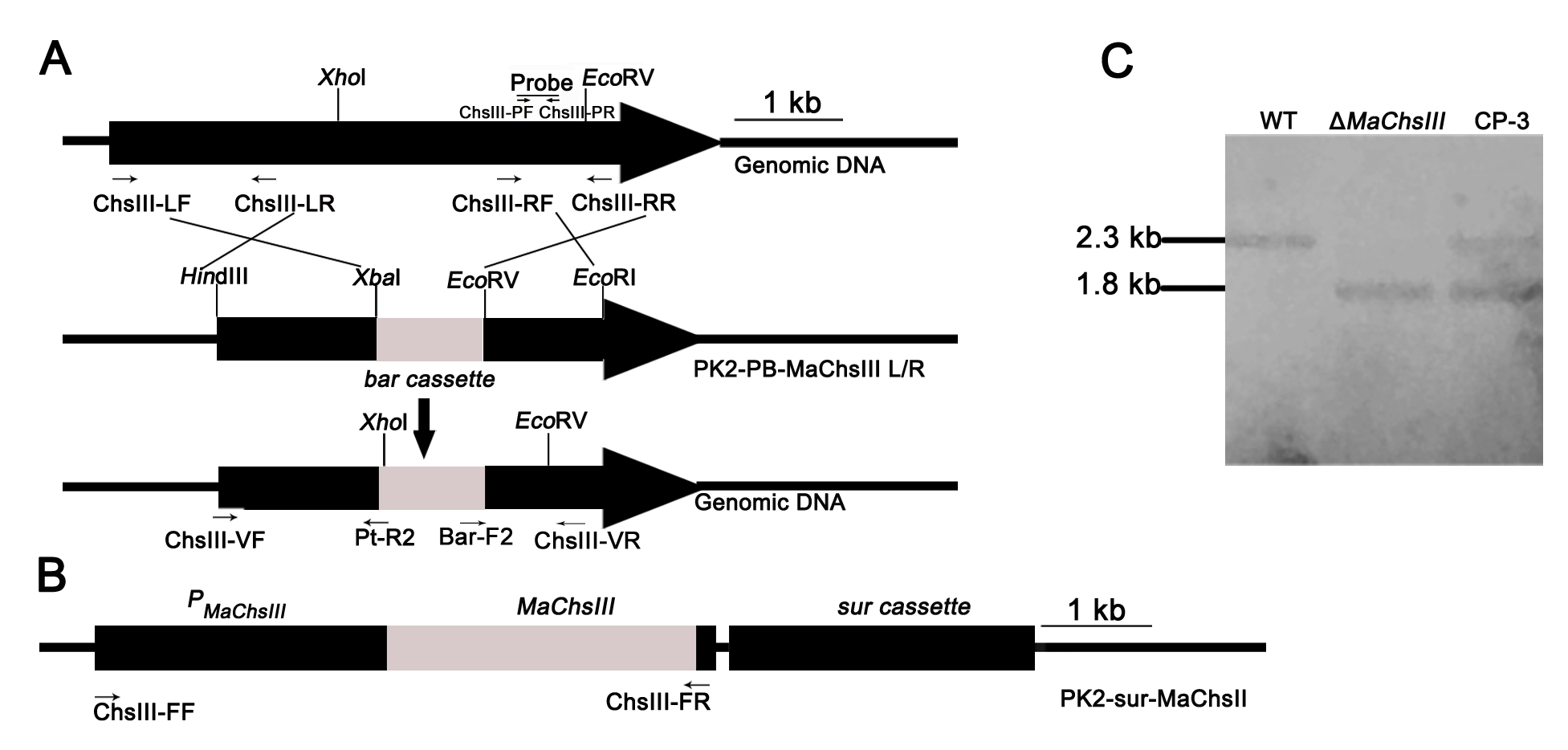

Supplement: S4 Fig — (A) Schematic illustration of the MaChsIII disruption in M. acridum. The probe was obtained by PCR using primers ChsIII-PF and ChsIII-PR. (B) Design of the MaChsIII complementation plasmid. (C) Southern blot analysis of the transformants hybridized by the probe. About 10 μg genomic DNA of WT, ΔMaChsIII, CP-3 was digested with EcoRV and XhoI. WT: the wild type; ΔMaChsIII: MaChsIII-disruption transformant; CP-3: MaChsIII-complementary transformant. (TIF) [file ppat.1007964.s006.tif]

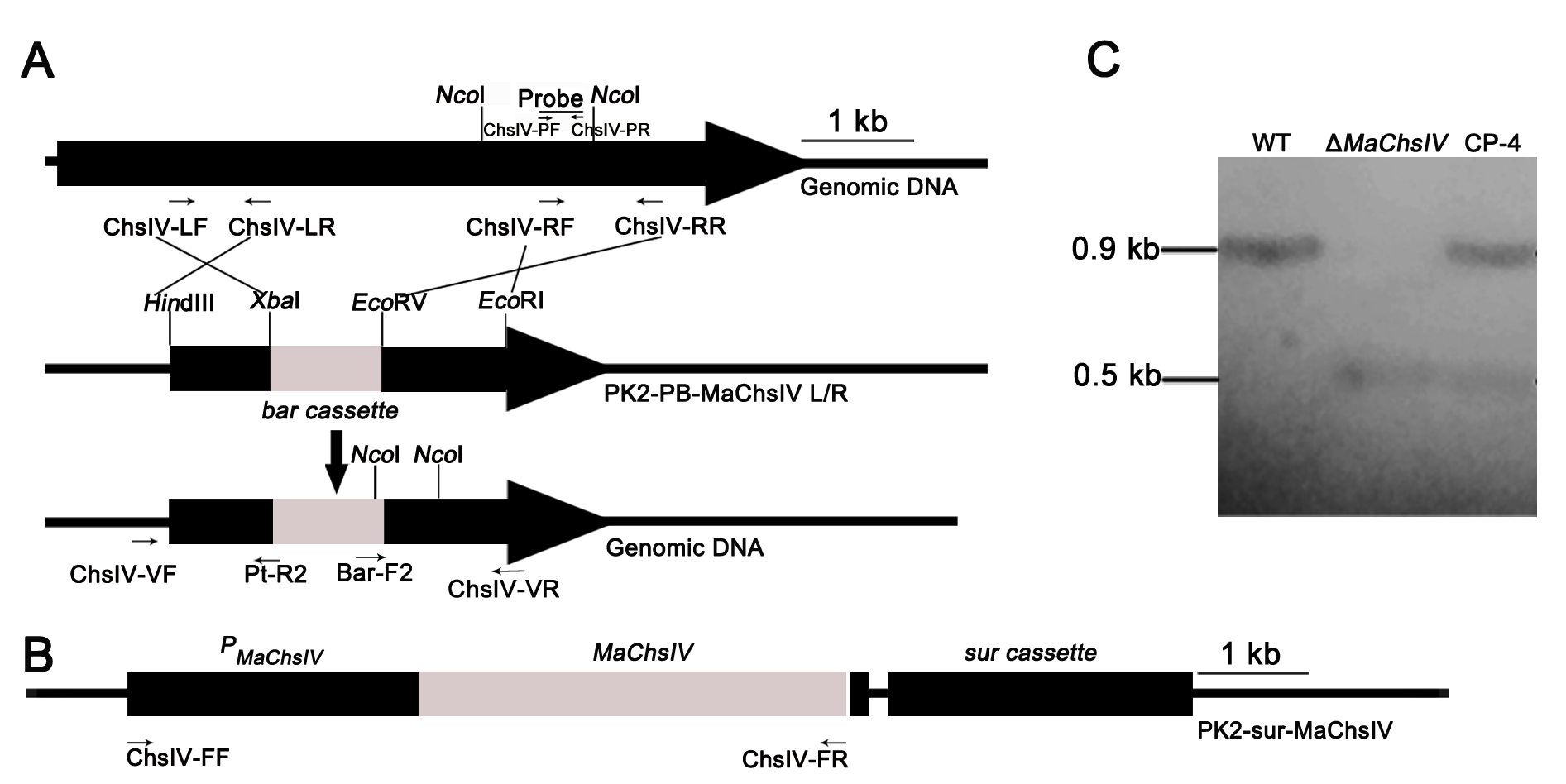

Supplement: S5 Fig — (A) Schematic illustration of the MaChsIV disruption in M. acridum. The probe was obtained by PCR using primers ChsIV-PF and ChsIV-PR. (B) Design of the MaChsIV complementation plasmid. (C) Southern blot analysis of the transformants hybridized by the probe. About 10 μg genomic DNA of WT, ΔMaChsIV, CP-4 was digested with NcoI. WT: the wild type; ΔMaChsIV: MaChsIV-disruption transformant; CP-4: MaChsIV-complementary transformant. (TIF) [file ppat.1007964.s007.tif]

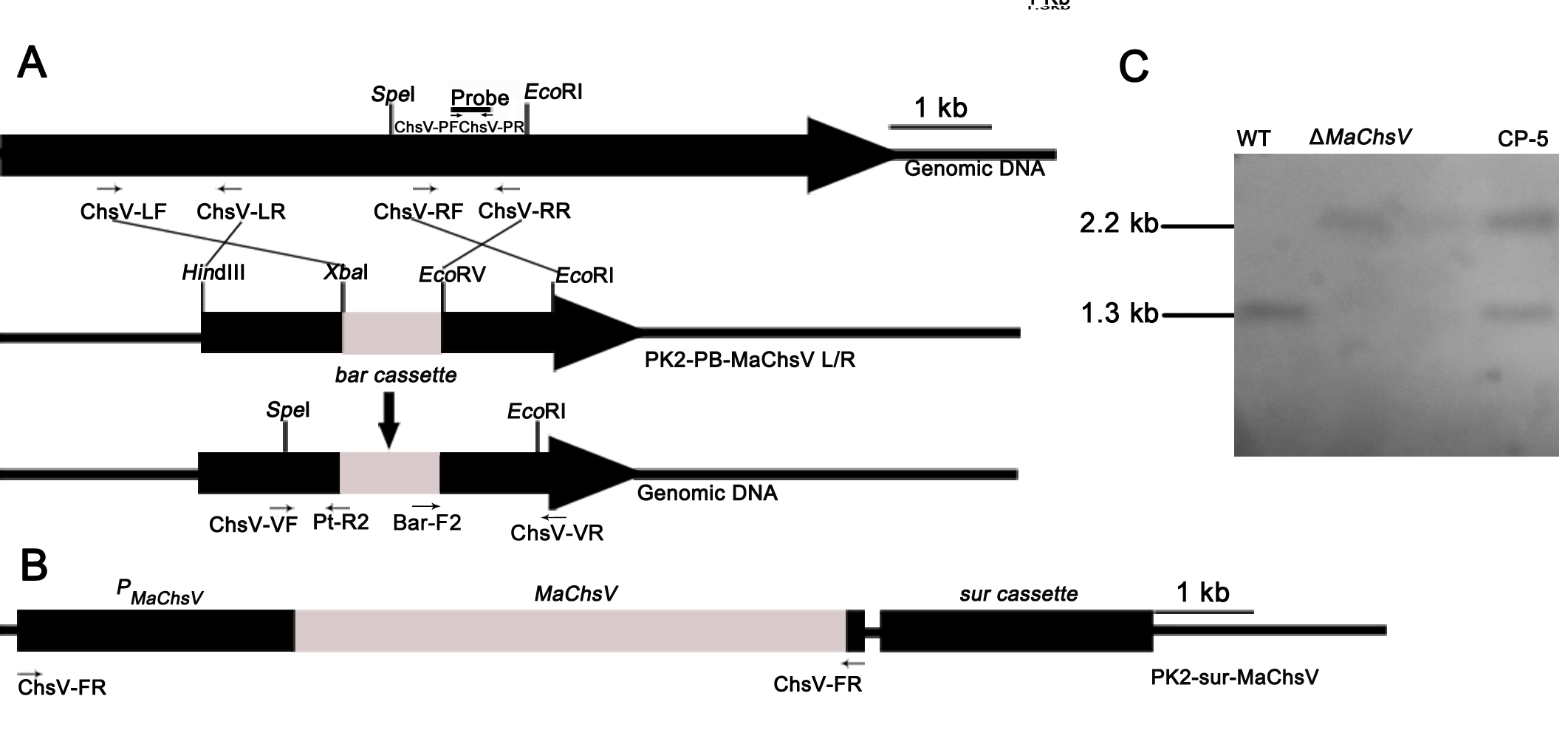

Supplement: S6 Fig — (A) Schematic illustration of the MaChsV disruption in M. acridum. The probe was obtained by PCR using primers ChsV-PF and ChsV-PR. (B) Design of the MaChsV complementation plasmid. (C) Southern blot analysis of the transformants hybridized by the probe. About 10 μg genomic DNA of WT, ΔMaChsV, CP-5 was digested with SpeI and EcoRI. WT: the wild type; ΔMaChsV: MaChsV-disruption transformant; CP-5: MaChsV-complementary transformant. (TIF) [file ppat.1007964.s008.tif]

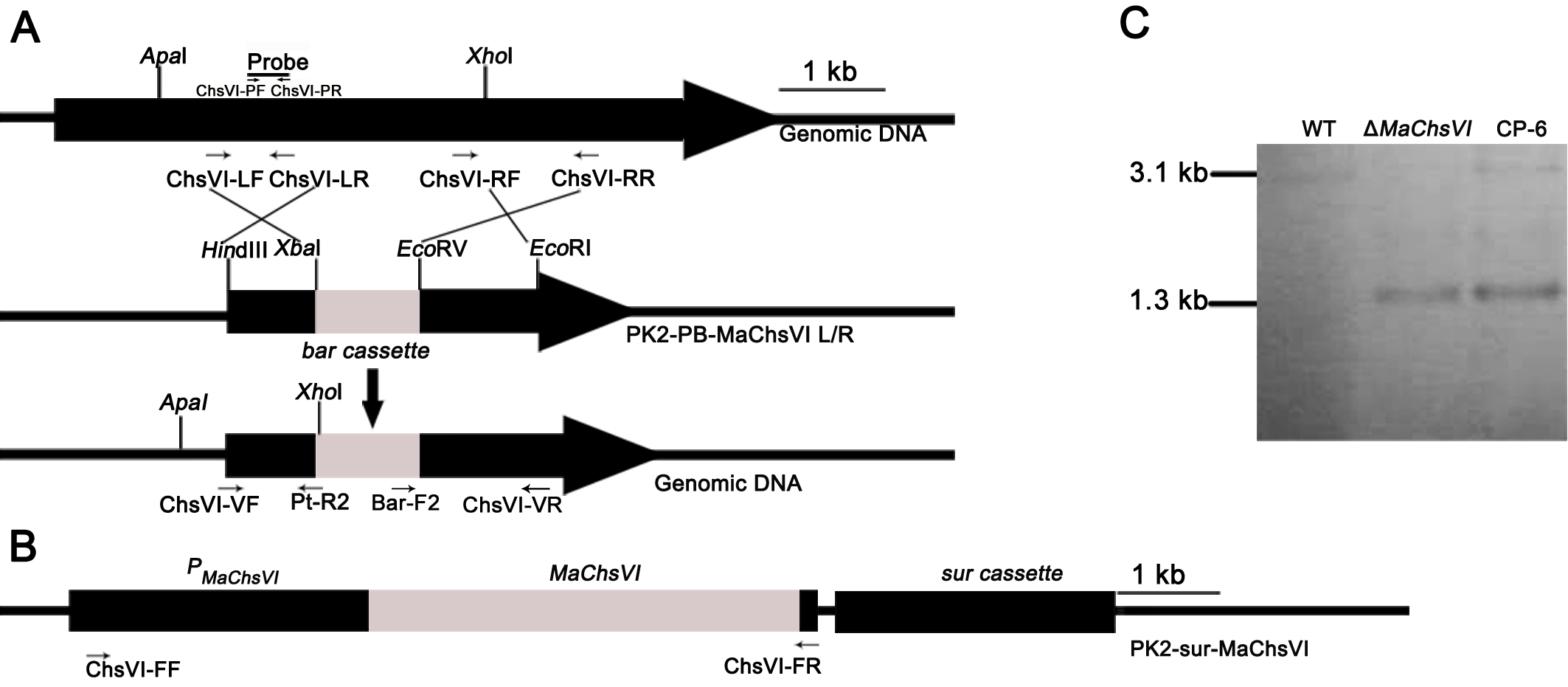

Supplement: S7 Fig — (A) Schematic illustration of the MaChsVI disruption in M. acridum. The probe was obtained by PCR using primers ChsVI-PF and ChsVI-PR. (B) Design of the MaChsVI complementation plasmid. (C) Southern blot analysis of the transformants hybridized by the probe. About 10 μg genomic DNA of WT, ΔMaChsVI, CP-6 was digested with ApaI and XhoI. WT: the wild type; ΔMaChsVI: MaChsVI-disruption transformant; CP-6: MaChsVI-complementary transformant. (TIF) [file ppat.1007964.s009.tif]

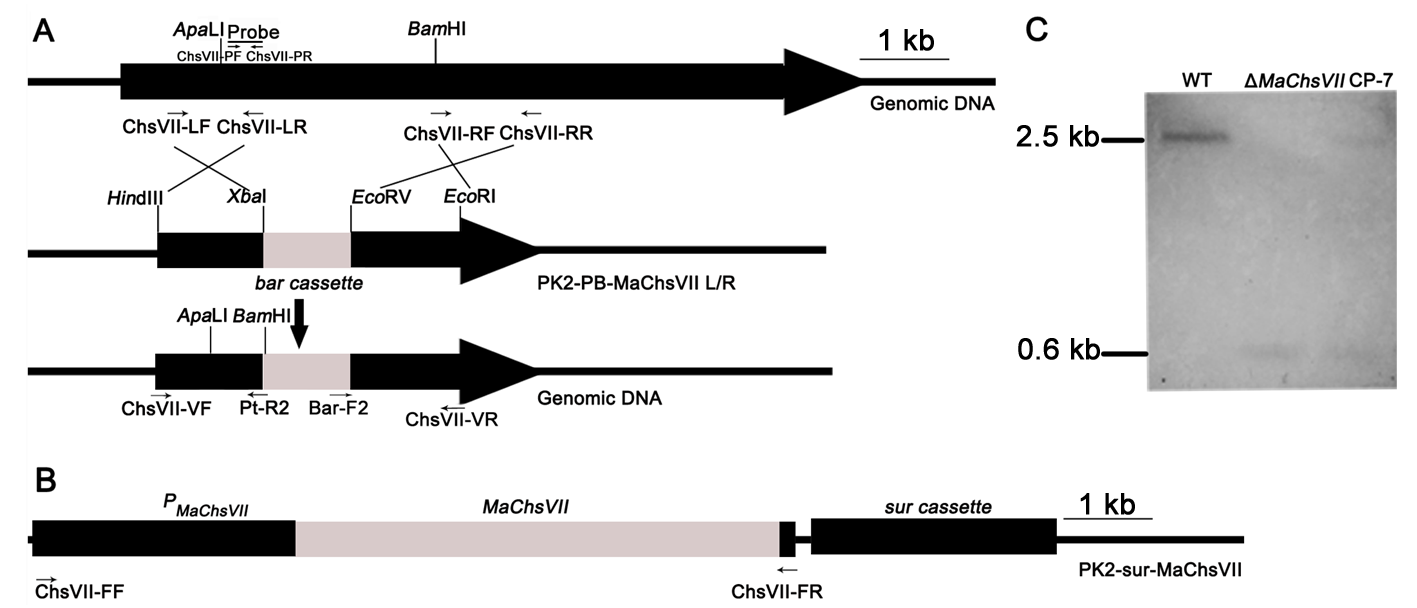

Supplement: S8 Fig — (A) Schematic illustration of the MaChsVII disruption in M. acridum. The probe was obtained by PCR using primers ChsVII-PF and ChsVII-PR. (B) Design of the MaChsVII complementation plasmid. (C) Southern blot analysis of the transformants hybridized by the probe. About 10 μg genomic DNA of WT, ΔMaChsVII, CP-7 was digested with ApaLI and XhoI. WT: the wild type; ΔMaChsVII: MaChsVII-disruption transformant; CP-7: MaChsVII-complementary transformant. (TIF) [file ppat.1007964.s010.tif]

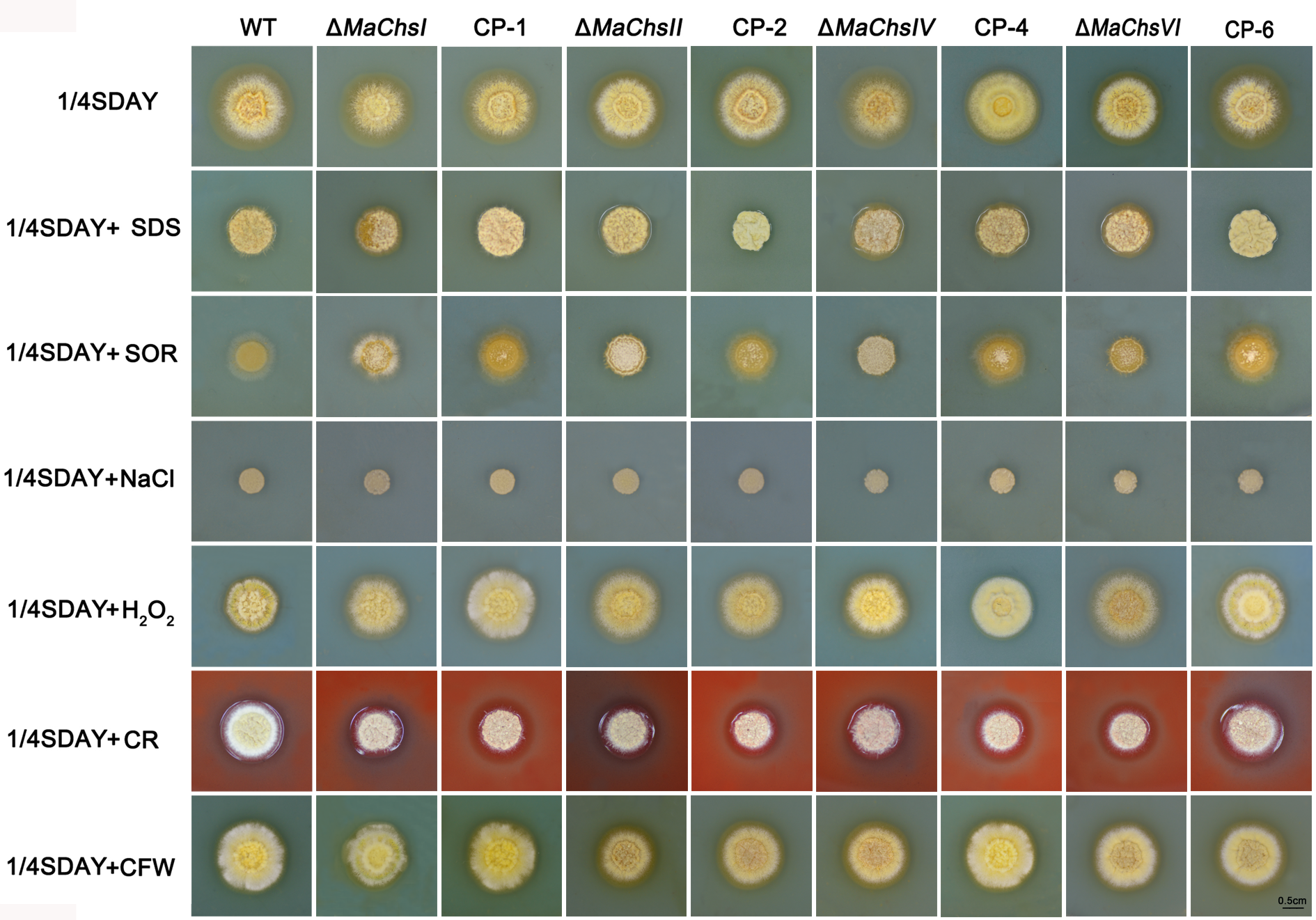

Supplement: S9 Fig — Colony morphology of the MaChs deletion mutants on 1/4 SDAY or 1/4 SDAY supplemented with 0.1% SDS, 1.5 mol l-1 Sorbitol, 0.5 mol l-1 NaCl, 500 μg ml-1 CR (Congo red), 50 μg ml-1 CFW (calcofluor white), 6 mmol l-1 H2O2 at 28°C. The fungal colonies were photographed after 5 d of incubation. Bar scale = 0.5 cm. (TIF) [file ppat.1007964.s011.tif]

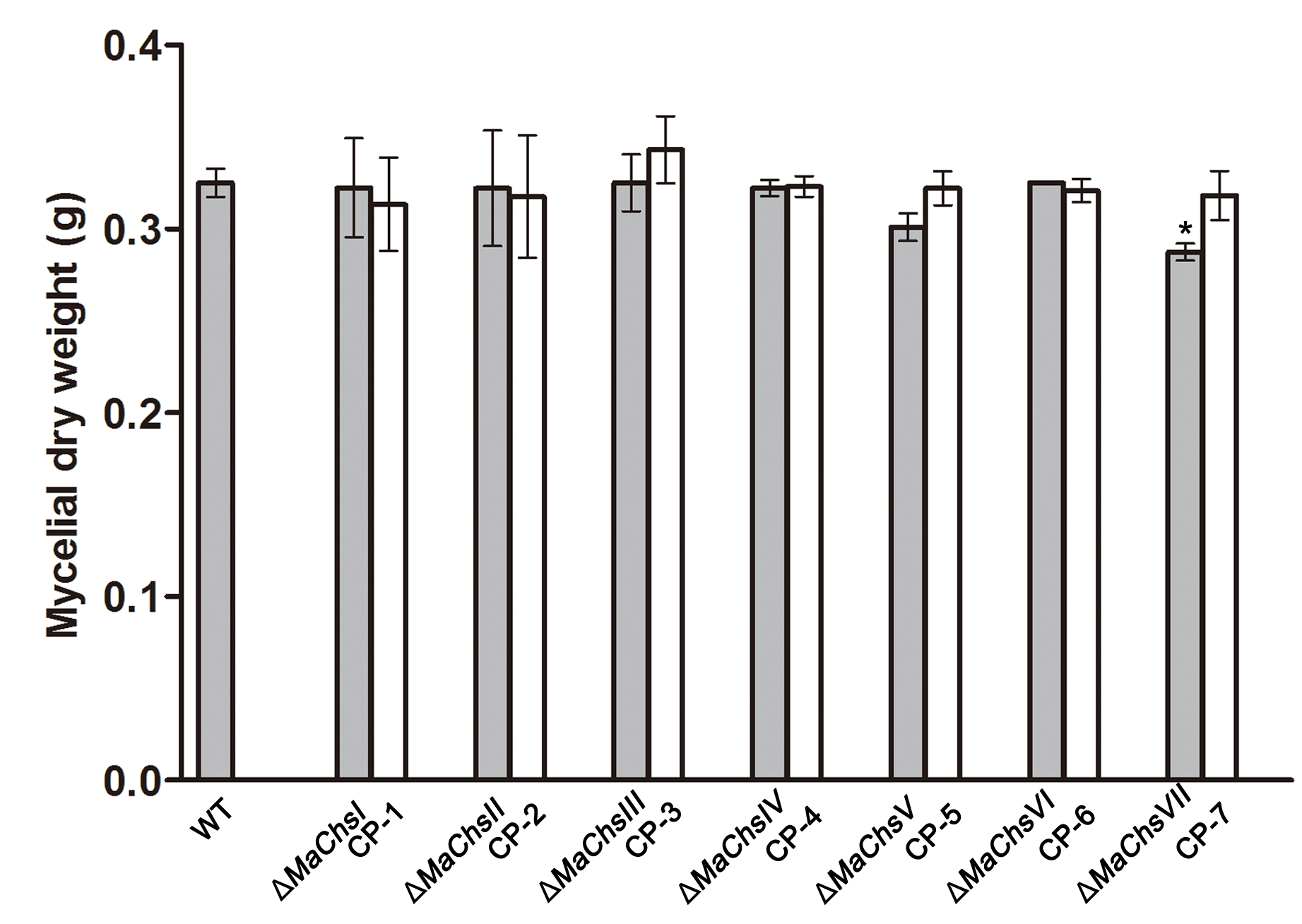

Supplement: S10 Fig — A single asterisk above bars denotes significant difference, P < 0.05. Error bars indicate standard errors of three trials. (TIF) [file ppat.1007964.s012.tif]

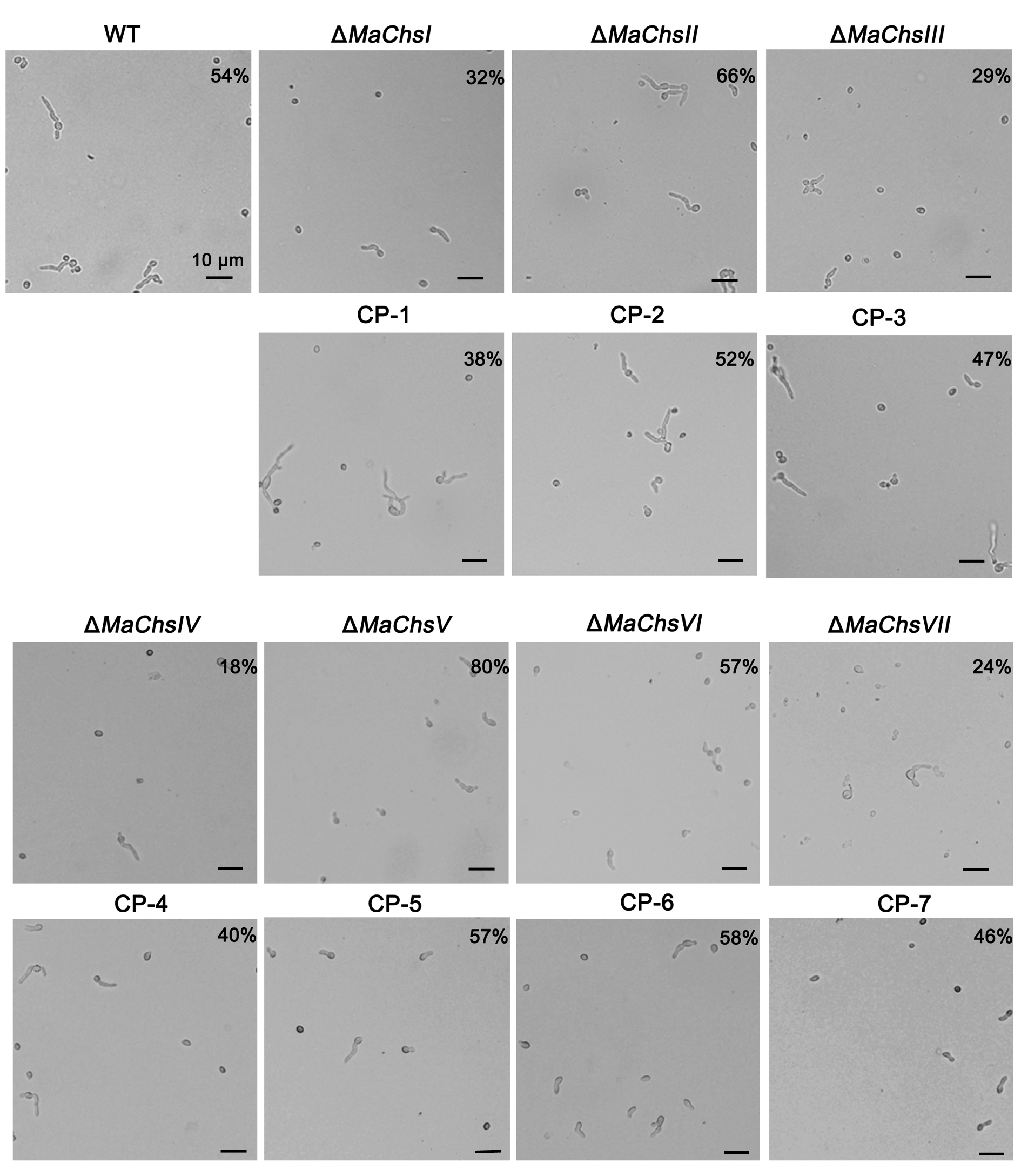

Supplement: S11 Fig — (TIF) [file ppat.1007964.s013.tif]

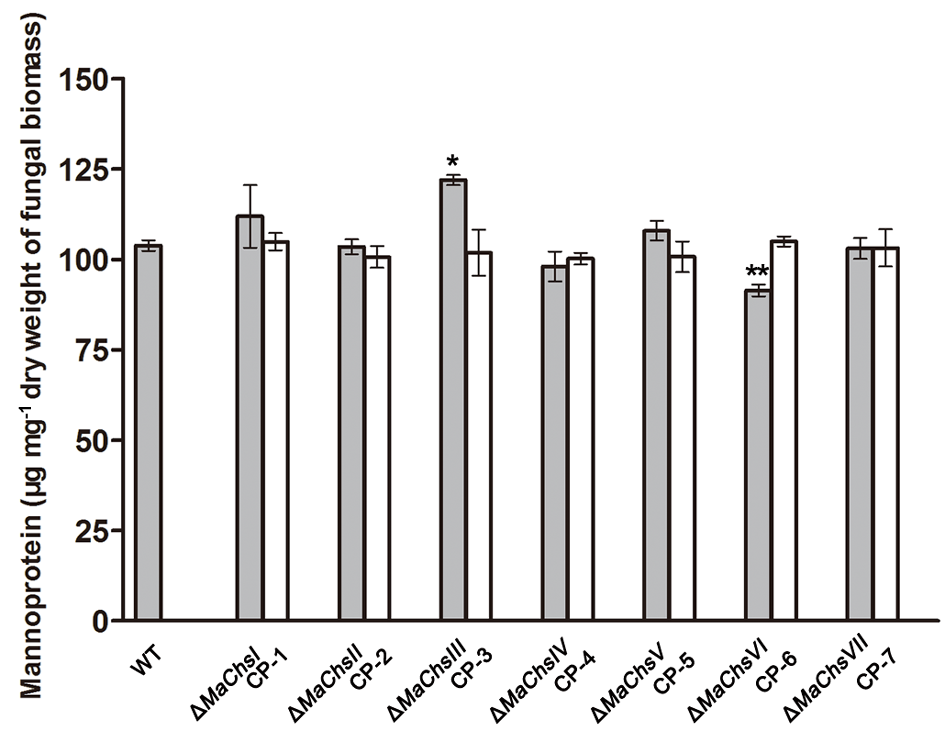

Supplement: S12 Fig — A single asterisk above bars denotes significant difference, P < 0.05; double asterisks above bars denote significant difference, P < 0.01. Error bars indicate standard errors of three trials. (TIF) [file ppat.1007964.s014.tif]

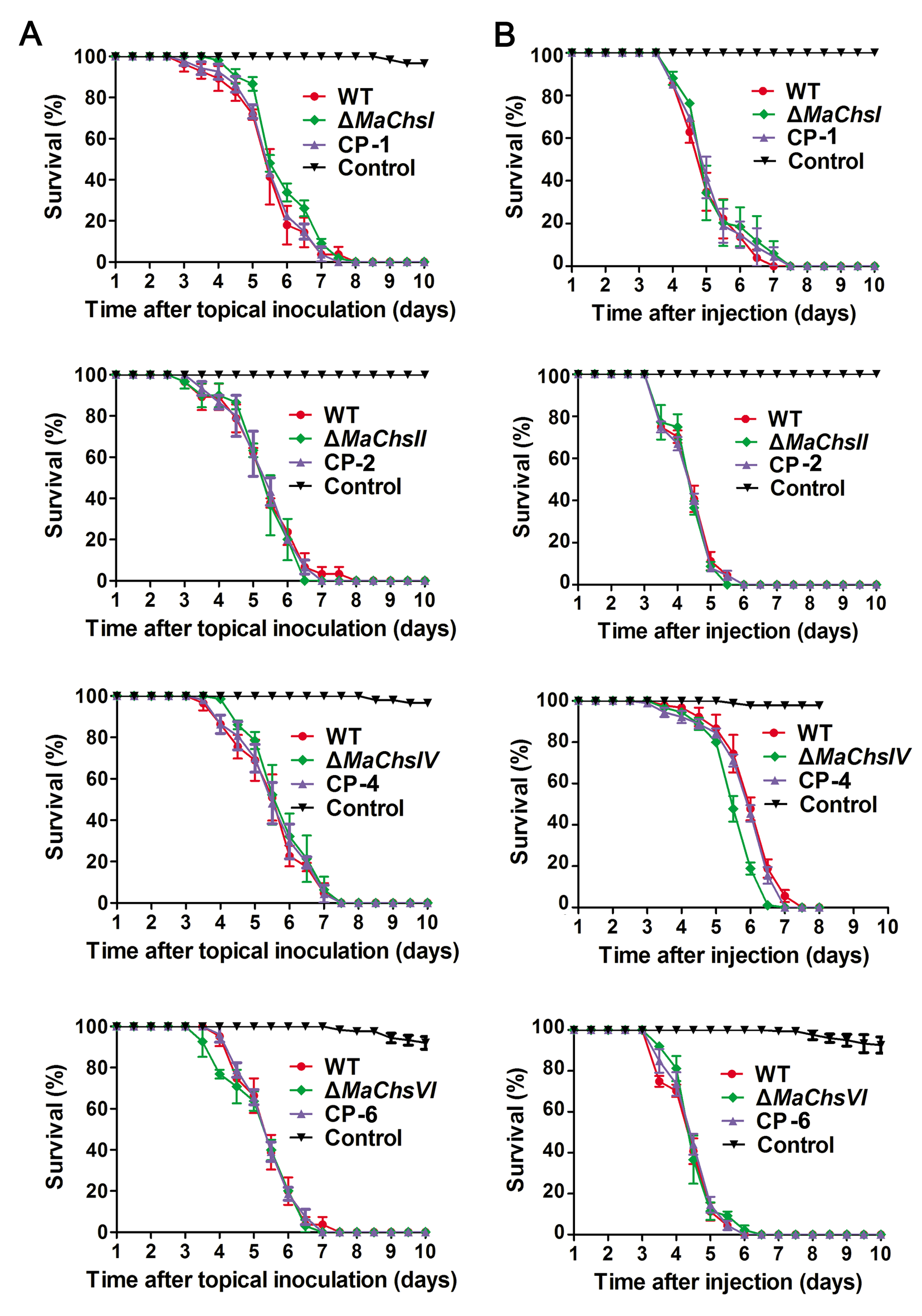

Supplement: S13 Fig — (A) Survival of locusts after topical inoculation with 5 μl Tween-80 (0.05%) containing 1×10^8^ conidia ml-1 of wild type and ΔMaChsI, ΔMaChsII, ΔMaChsIV, ΔMaChsVI mutants. (B) Survival of locusts after injection with 5 μl sterile water containing 1×10^6^ conidia m-1 of wild type and ΔMaChsI, ΔMaChsII, ΔMaChsIV, ΔMaChsVI mutants. Error bars indicate standard errors of three trials. (TIF) [file ppat.1007964.s015.tif]

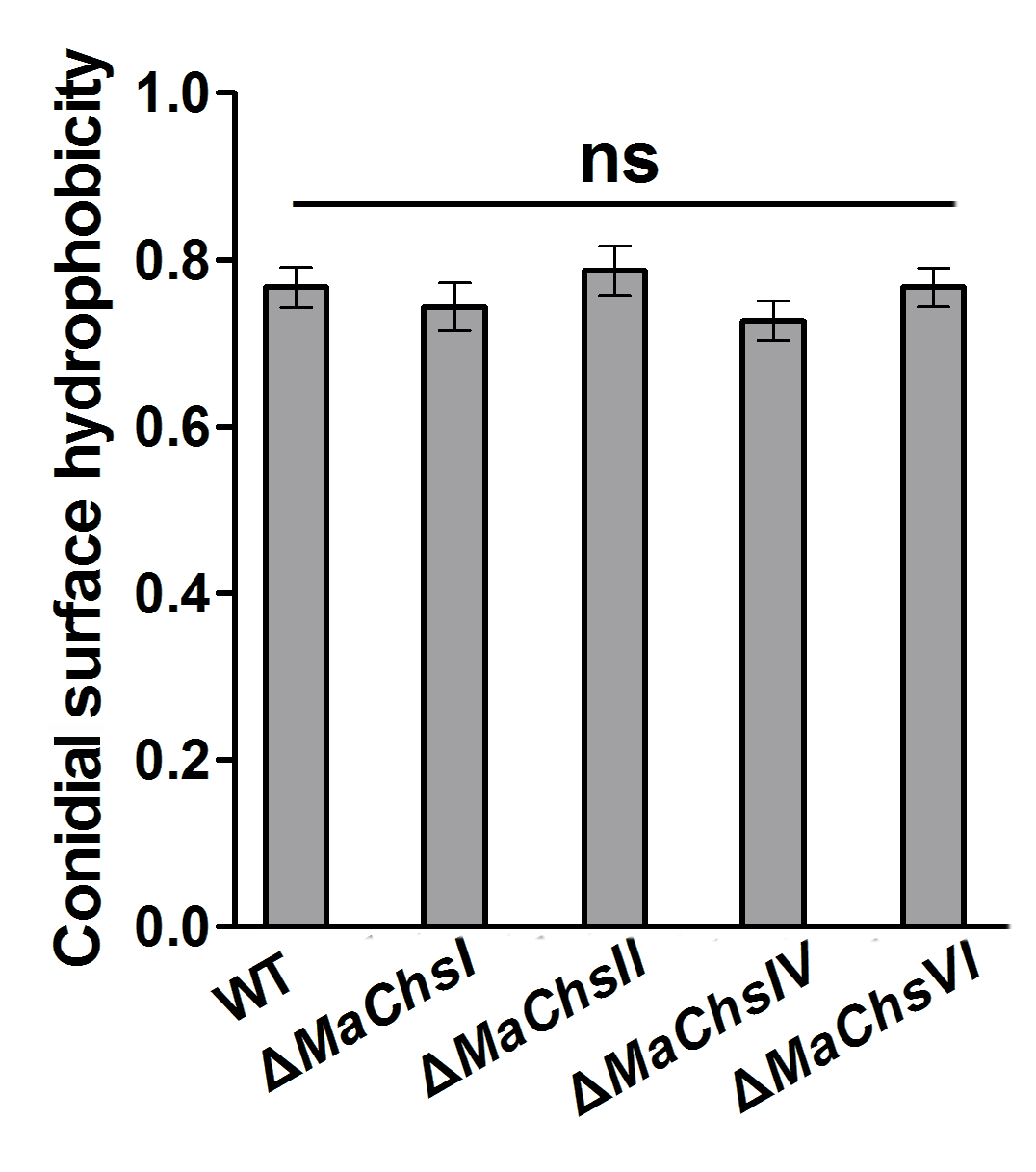

Supplement: S14 Fig — ns indicates no significant difference, P > 0.05. Error bars indicate standard errors of three trials. (TIF) [file ppat.1007964.s016.tif]
